# HDAC1 modulates sepsis-induced immunosuppression by driving the exhaustion of CD8^+^ T cells

**DOI:** 10.1172/jci.insight.197224

**Published:** 2026-02-23

**Authors:** Liu Di, Jiang-bo Fan, Rui Wang, You Li, Wan-da Bi, Si-yuan Huang, Heng-hai Nie, Xi-feng Feng, Hua-cai Zhang, Juan Du, Xiao-fei Huang, An-yong Yu, Zhe Xu, Fei Xia, Jian-xin Jiang, Shuang-shuang Dai, Xiang Xu, Zhen Wang, Ling Zeng

**Affiliations:** 1 Department of Trauma Medical Center, and; 2 Department of Stem Cell & Regenerative Medicine, State Key Laboratory of Trauma and Chemical Poisoning, Daping Hospital, Army Medical University, Chongqing, China.; 3 Department of Biochemistry and Molecular Biology, School of Basic Medicine, Army Medical University, Chongqing, China.; 4 Department of Intensive Care Unit, Daping Hospital, State Key Laboratory of Trauma and Chemical Poisoning, Army Medical University, Chongqing, China.; 5 Department of Emergency, Affiliated Hospital of Zunyi Medical University, Zunyi, China.; 6 Department of Emergency, the Affiliated Hospital of Guizhou Medical University, Guizhou Medical University, Guiyang, China.

**Keywords:** Immunology, Infectious disease, Inflammation, Adaptive immunity

## Abstract

Sepsis, a systemic inflammatory response to infection, remains a leading cause of mortality in intensive care units, with sepsis-induced immunosuppression being a critical pathophysiological process. In this study, we investigated the role of histone deacetylase 1 (HDAC1) in sepsis-induced CD8^+^ T cell exhaustion, a key driver of immunosuppression. Clinical analyses of patients with sepsis revealed that reduced peripheral blood lymphocyte levels, particularly CD8^+^ T cell depletion, strongly correlated with worsened outcomes. In a murine sepsis model, single-cell RNA-Seq revealed a significant decrease in the proportion of CD8^+^ T cells and an increase in the proportion of exhausted CD8^+^ T cells in mouse lungs. Adoptive transfer of CD8^+^ T cells effectively reduced sepsis mortality by preserving organ function. We further demonstrated that HDAC1 expression was significantly upregulated in CD8^+^ T cells from patients with sepsis. In vitro studies showed that HDAC1 inhibition preserved CD8^+^ T cell function by maintaining T cell activity and reducing the expression of inhibitory molecules such as PD-1. Pharmacological inhibition of HDAC1 reduced mortality and reversed CD8^+^ T cell exhaustion by restoring the balance between activator protein-1 (AP-1) and nuclear factor of activated T cells (NFAT). Additionally, we found that HDAC1 directly interacted with NFAT1, promoting its nuclear translocation and further enhancing the expression of inhibitory molecules. Our findings highlight HDAC1 as a potential therapeutic target for sepsis-induced immunosuppression. By elucidating the molecular mechanisms underlying HDAC1-mediated immunosuppression, we have provided potential strategies for developing immunomodulatory therapies for the treatment of sepsis.

## Abstract 2

Sepsis impairs immunity by exhausting CD8+ T cells; blocking HDAC1 restores immune function and reduces mortality, suggesting a new therapeutic strategy.

## Graphical abstract

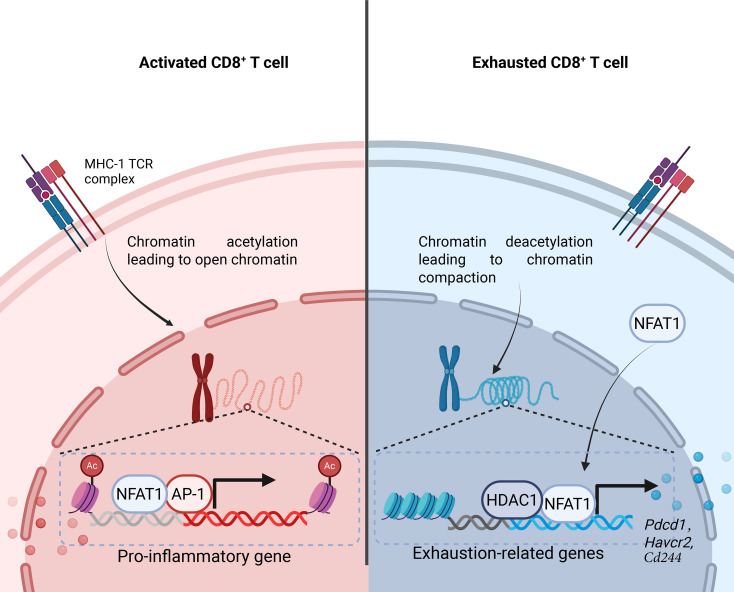

## Introduction

Sepsis is a lethal, systemic multiorgan dysfunction caused by infection and resulting from an uncontrollable inflammatory response. Despite advances in intensive care, the mortality rates associated with sepsis and septic shock remain high. The in-hospital mortality rate of patients with sepsis is 10% to 20%. Septic shock, a subtype of sepsis that is characterized by severe cardiovascular abnormalities, has a 40% to 50% mortality rate (1). The immunopathology of sepsis is characterized by a biphasic response: initial hyperinflammation followed by profound immunosuppression. Although early innate immune clearance can restore homeostasis, persistent immune dysfunction contributes to secondary infections, which are now recognized as major drivers of sepsis-related death (2, 3). During sepsis-induced immunosuppression, macrophages are overactivated, DCs exhibit defects in antigen presentation or even undergo apoptosis, bone myeloid–derived suppressor cells (MDSCs) expand, and most lymphocytes except Tregs become dysfunctional and can even undergo cell death (4). These changes explain why therapeutic strategies aimed at blocking hyperinflammation, such as glucocorticoids or cytokine inhibitors, have largely failed in clinical trials (5, 6).

T lymphocytes, particularly CD8^+^ T cells, are central to adaptive immunity and play a pivotal role in pathogen clearance. Hotchkiss et al. reported extensive T cell death in the lungs and spleens of patients with sepsis and noted significant upregulation of inhibitory receptors, including PD-1, PD-L1, and CD25, on T cells (7, 8). The counts and ratios of CD4^+^ T cells and CD8^+^ T cells can serve as preliminary indicators for monitoring the immune status of patients with sepsis (9). Our prior work demonstrated increased TIM3 expression on CD4^+^ T cells and functional suppression mediated by HMGB1, but whether CD8^+^ T cells undergo similar regulatory changes remains unclear (6). Unlike infections such as malaria, where CD8^+^ T cell activation is enhanced (10), sepsis is associated with reduced CD8^+^ T cell counts and impaired cytotoxic function, which strongly correlates with secondary infections and mortality (11–13).

The imbalance between the proinflammatory immune response and antiinflammatory immune response is the pathophysiological basis for sepsis-induced immunosuppression, and epigenetic modifications regulate this balance (14). Class I histone deacetylase (HDAC1) is a histone deacetylase that typically mediates histone deacetylation on chromatin, causing the chromatin structure to condense, thereby silencing gene transcription (15, 16). In humans, HDACs are divided into 2 families on the basis of their different catalytic mechanisms: the HDAC1–11 family and the sirtuin family (17). Many studies have focused on the therapeutic efficacy of HDAC inhibitors (HDACis) in sepsis. Butyrate is a short-chain fatty acid and a potent HDACi that increases the survival rate of septic mice and reduces intestinal injury caused by sepsis (18). Valproic acid is a class I HDACi that can alleviate sepsis-induced myocardial dysfunction by accelerating autophagy (19). Although HDACis have shown promise in modulating immune responses during sepsis, the molecular link between HDAC and CD8^+^ T cell exhaustion in sepsis has not been established.

Here, we integrated large-scale clinical data, single-cell transcriptomics, bulk RNA-Seq, and mechanistic experiments to characterize CD8^+^ T cell dysfunction during sepsis. We identified HDAC1 as a key epigenetic driver of CD8^+^ T cell exhaustion through interaction with nuclear factor of activated T cells (NFAT1) and disruption of the transcriptional balance of activator protein-1 (AP-1) and NFAT. Pharmacological inhibition of HDAC1 restored T cell function and improved survival in sepsis models, highlighting HDAC1 as a promising therapeutic target for sepsis-induced immunosuppression.

## Results

### The percentage of peripheral blood lymphocytes, particularly the proportion of CD8^+^ T cells, serves as a key biomarker for the progression of sepsis.

**Figure 1:**
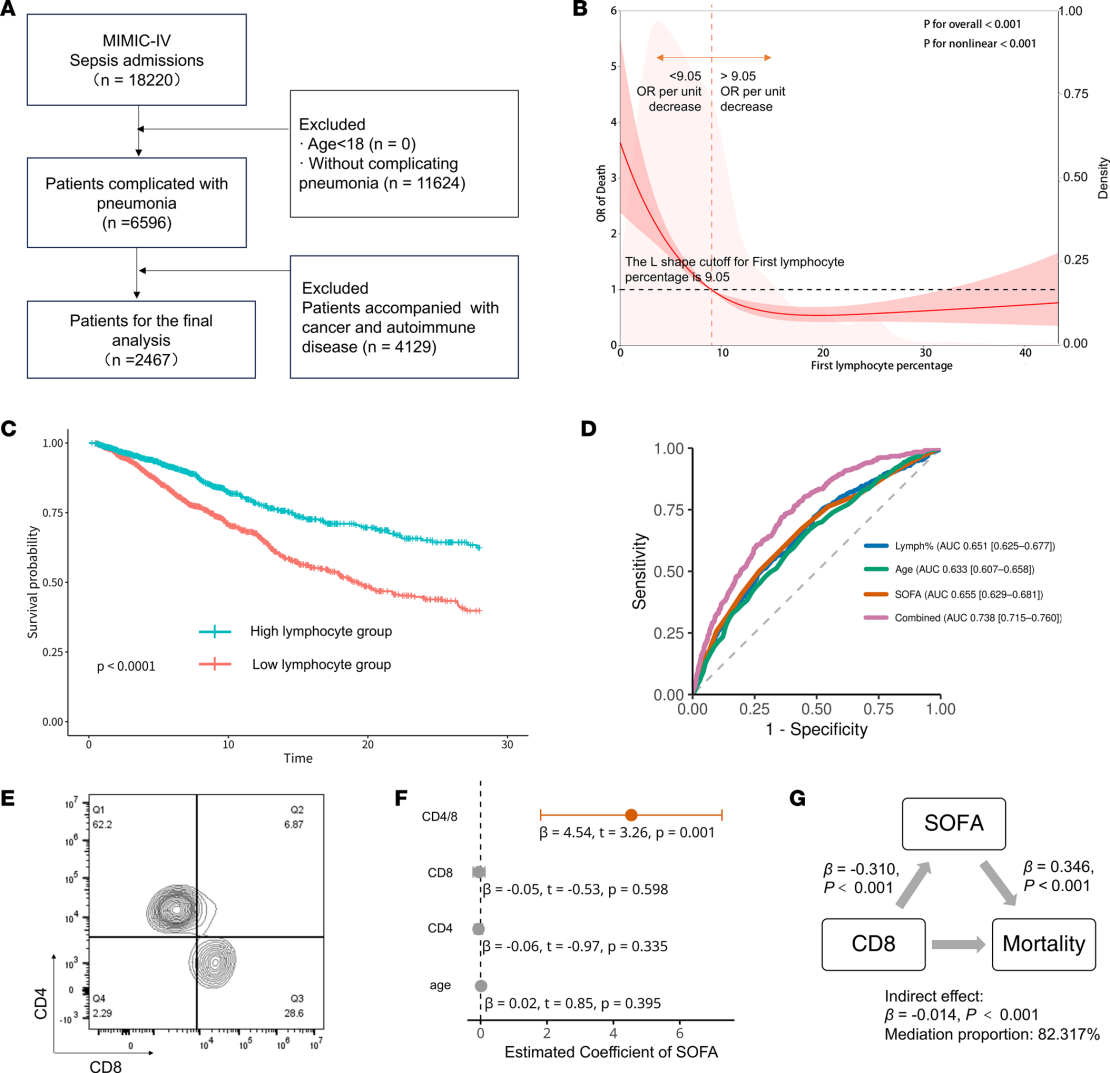
Associations between lymphocyte counts and the CD4/CD8 ratio and prognosis of patients with sepsis. (**A**) Flowchart showing selection of patients from MIMIC-IV database: 18,220 sepsis admissions were initially identified; after excluding patients without pneumonia (*n* = 11,624) and those with cancer or autoimmune diseases (*n* = 4,129), 2,467 patients were included in the final analysis. (**B**) Restricted cubic spline (RCS) for relationships between initial lymphocyte percentage after admission and 28-day ICU mortality in MIMIC-IV cohort. Solid red line is estimated ORs with 95% CI (red area); vertical dashed line is L-shaped cutoff (9.05%); solid pink curves are fraction of population with different lymphocyte percentages. *P* < 0.001 for overall association and nonlinearity. (**C**) Kaplan-Meier survival curves comparing 28-day survival between patients with sepsis with low versus high lymphocyte percentage, stratified by median value at ICU admission. Log-rank test was used to assess differences between groups (*P* < 0.0001). (**D**) ROC curves comparing predictive performance of initial lymphocyte percentage (AUC = 0.6469), SOFA score (AUC = 0.6521), and age (AUC = 0.6254) for 28-day mortality in patients with sepsis. AUC with 95% CIs is indicated. AUC comparisons were performed using DeLong’s test. (**E**) Representative flow cytometry contour plot showing CD4^+^ and CD8^+^ T cell subsets gated on live CD3^+^ lymphocytes from peripheral blood of patients with sepsis. Quadrants indicate proportions of CD4^+^ (upper left), CD8^+^ (lower right), double-negative (lower left), and double-positive (upper right) T cell populations. (**F**) Multivariate linear regression analysis assessing factors associated with SOFA score. Variables included CD4/CD8 ratio, CD8^+^ T cell proportion, CD4^+^ T cell proportion, and age. *β* coefficients with 95% CIs and *P* values are shown. (**G**) Mediation analysis illustrating indirect effect of CD8^+^ T cell proportion on 28-day mortality through SOFA score. Total, direct, and indirect effects (*β* coefficients) with corresponding *P* values shown. Indirect effect significance assessed using Bayesian approach (1,000 iterations).

We conducted a retrospective analysis to assess the prognostic value of the peripheral blood lymphocyte percentage in patients with sepsis with concomitant pneumonia admitted for more than 7 days using the Medical Information Mart for Intensive Care IV (MIMIC-IV database) (20). We specifically focused on pneumonia-related sepsis because pulmonary infection represents the leading source of sepsis and is strongly associated with profound immune dysregulation and susceptibility to secondary infections. Restricting the cohort to this subgroup minimized heterogeneity due to different infection sites and enhanced biological relevance for subsequent lung-focused mechanistic studies. After excluding patients with cancer or autoimmune diseases, a total of 2,467 patients were included in the analysis (Figure 1A). The exposure variable was defined as the first peripheral blood lymphocyte percentage measured within 24 hours after ICU admission. The median lymphocyte percentage was 9.00% (IQR 5.30–14.00). Baseline characteristics stratified by the median value are shown in Table 1. Patients with lymphocyte percentages below the median were older (65.58 ± 16.77 vs. 61.95 ± 17.31 years, *P* < 0.001), had higher Sequential Organ Failure Assessment (SOFA) scores (7.41 ± 3.94 vs. 6.55 ± 3.76, *P* < 0.001), and experienced longer ICU stays (10.06 ± 9.64 vs. 9.14 ± 9.26 days, *P* = 0.016) compared with those above the median. Most notably, the lower lymphocyte percentage group exhibited substantially greater ICU mortality (31.47% vs. 15.64%, *P* < 0.001), whereas the sex distribution was not significantly different between the groups (*P* = 0.656) (Table 1). Consistent with these differences, Kaplan-Meier survival curves stratified by the prespecified median cutoff (9.00%) showed significantly lower survival in the low-lymphocyte group throughout the follow-up period, with early and persistent separation of the curves (log-rank *P* < 0.0001; Figure 1C). The restricted cubic spline regression adjusted for age, sex, and SOFA score revealed an L-shaped association between lymphocyte percentage and 28-day mortality; beyond 9.05%, the incremental mortality risk was substantially attenuated (Figure 1B). This nonlinear relationship underscores the clinical utility of monitoring the lymphocyte percentage as an early warning threshold for patient prognosis. Predictive performance analysis showed that lymphocyte percentage alone yielded an AUC of 0.647, comparable to SOFA (AUC = 0.652) and superior to age (AUC = 0.625). Importantly, combining lymphocyte percentage with SOFA significantly improved prediction (AUC = 0.738, *P* < 0.001) (Figure 1D).

To further investigate the relationships between T lymphocyte subsets and disease severity, prospective T cell data were collected from 140 patients with sepsis admitted to the ICU for more than 7 days (Supplemental Table 1; supplemental material available online with this article; https://doi.org/10.1172/jci.insight.197224DS1). Multivariable linear regression analysis revealed that the CD4^+^/CD8^+^ T cell ratio measured by flow cytometry (Figure 1E) in peripheral blood was positively associated with the SOFA score (*β* = 4.88, *P* < 0.001), whereas neither CD4^+^ T cell percentage, CD8^+^ T cell percentage, nor age exhibited significant associations after covariate adjustment (all *P* > 0.26) (Figure 1F). To delineate the role of T cell subsets in sepsis progression, Bayesian causal mediation analysis was performed with CD8 percentage as the exposure, SOFA score as the mediator, and ICU mortality as the outcome. CD8^+^ T cell percentage was significantly associated with lower SOFA scores (*β* = –0.310; *P* < 0.001), and the indirect effect of CD8^+^ T cell percentage on mortality via SOFA was statistically significant (*β* = –0.014; 95% CI: –0.024 to –0.003; *P* < 0.001), accounting for 82.317% of the total effect, whereas the direct effect was nonsignificant (Figure 1G and Supplemental Table 2). These findings indicate that CD8^+^ T cell depletion may influence clinical outcomes primarily through its contribution to organ failure severity. In contrast, mediation analysis using CD4^+^ T cell percentage as the exposure did not reveal significant indirect or direct effects on mortality (indirect effect *β* = 0.001, *P* = 0.306; mediation proportion 37.304%; Supplemental Table 3), suggesting that CD4^+^ T cell dynamics play a limited role in determining outcomes through the SOFA score. These findings underscore the prognostic relevance of CD8^+^ T cell dynamics in sepsis and highlight the SOFA score as a mechanistic link between immune dysregulation and clinical outcomes.

### Single-cell sequencing analysis revealed a significant decrease in CD8^+^ T cells and an increase in the proportion of exhausted CD8^+^ T cells in the lungs of septic mice.

**Figure 2:**
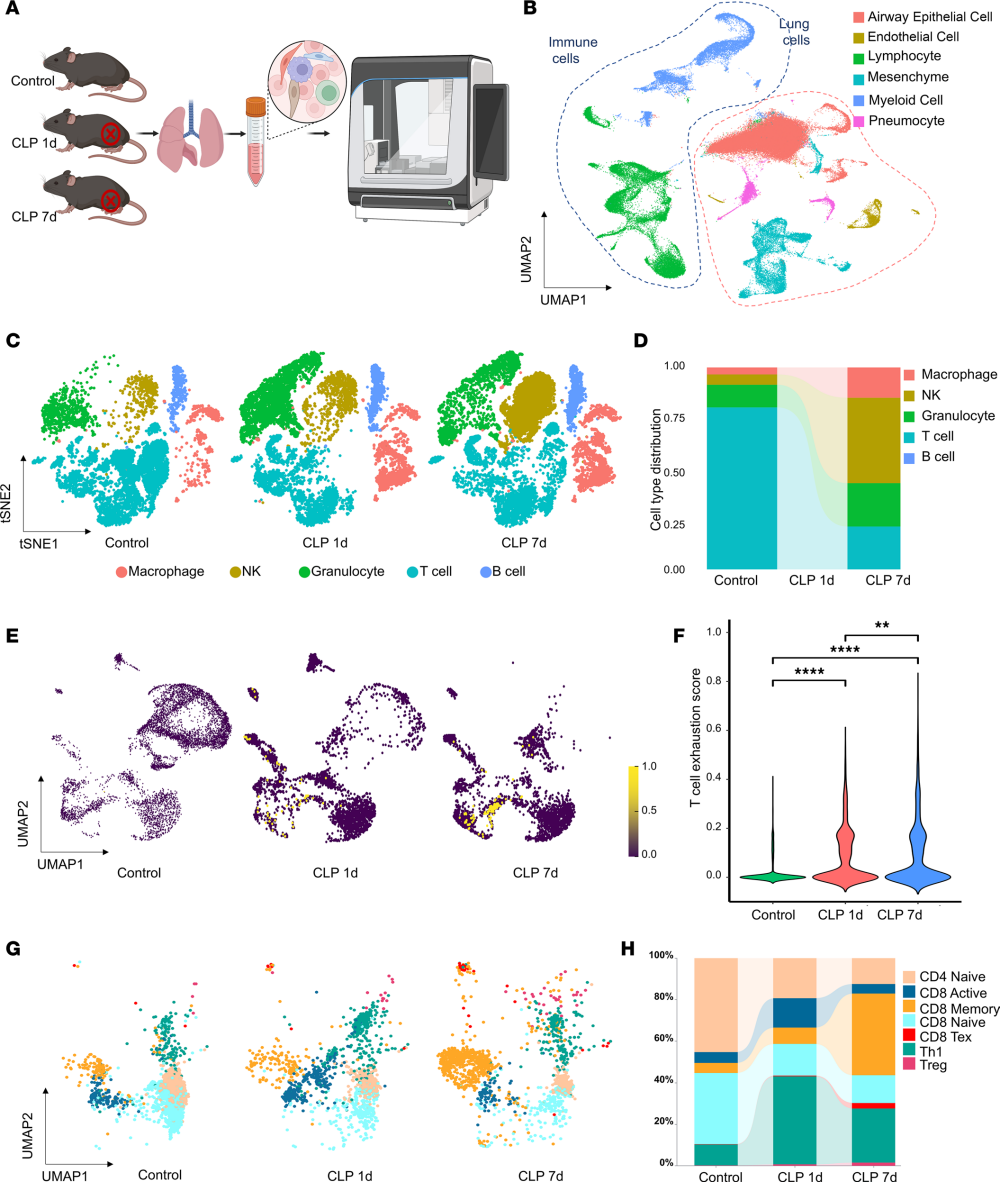
Single-cell atlas of pulmonary immune cells during sepsis progression. (**A**) Schematic diagram showing scRNA-Seq pipeline of murine pulmonary immune cells during sepsis progression. (**B**) UMAP plot of scRNA-Seq data from lung tissue. (**C**) UMAP plots of pulmonary immune cells from healthy or CLP mice at different time points after CLP, annotated by cell type. Each time point includes data from either 3 mice (1 day or 7 days) or 2 mice (healthy controls). (**D**) Stacked bar chart showing the percentages of different immune cell types at various time points. (**E**) Feature plots illustrating the distribution of T cell exhaustion gene signature scores in T cells in the healthy control, CLP-1d, and CLP-7d groups. The Mann-Whitney *U* test was employed to calculate signature scores. The color intensity corresponds to the magnitude of the signature score, with yellow dots representing higher exhaustion scores. (**F**) Violin plot displaying the exhaustion scores of T cells across different groups (**P < 0.01, ****P < 0.0001; Kruskal-Wallis test followed by Dunn’s multiple-comparison test). (**G**) UMAP plots illustrating the temporal changes in T cell subtypes using Project TIL for detailed T cell subtype classification. (**H**) Stacked bar chart depicting the percentages of different T cell subtypes in the indicated groups. CD8 Tex, CD8 exhausted T cells.

Lung infections, particularly severe pneumonia, are the most common causes of sepsis, often leading to acute lung injury and acute respiratory distress syndrome. The lungs are highly susceptible to sepsis-induced damage, and respiratory failure is among the most severe complications of sepsis. Additionally, sepsis triggered by extrapulmonary factors, such as pancreatitis, systemic infections, trauma, and burns, can also result in pulmonary damage (21, 22). To explore the mechanisms of immune cell dysfunction in the context of sepsis, we established a sepsis murine cecal ligation and puncture (CLP) model and a second-hit model of sepsis. The second-hit model of sepsis more accurately reflects susceptibility to secondary infections and the immunosuppressive state (6). We then performed scRNA-Seq to comprehensively profile lung immune cells. The lungs of healthy control mice at day 1 and day 7 after CLP were analyzed. A total of 132,094 cells were selected for further analysis after quality control, which generated, on average, approximately 20,967 mapped reads and 2,268 genes per cell (Figure 2A). As expected, unbiased clustering analysis revealed multiple clusters of pulmonary immune cells, which included macrophages (Ms4a6c/4a/6d and Ctss), neutrophils (S100a8/a9 and Retnlg), NK cells (Nkg7 and Klrb1c/k1c), T cells (Cd3e/d/g, Itk and Camk4), and B cells (Igkc and Cd79a/b) (Figure 2, B and C, and Supplemental Figure 1). Compared with those in healthy controls, CLP induced significant changes in the immune cell composition, and the proportions of T lymphocytes in the lungs decreased during the development of CLP-induced sepsis (Figure 2, C and D). We further assessed T cell states and subsets. The exhaustion scores of all the T cells revealed that the highest level of exhaustion was observed at 7 days after CLP (Figure 2, E and F). This temporal pattern suggests a progressive increase in T cell exhaustion as sepsis progresses. Furthermore, by projecting T cell subtypes onto a reference dataset of murine T cells (23), we identified a significant decrease in the proportion of CD8^+^ T cells at 7 days after CLP. Concurrently, there was a marked increase in the proportion of exhausted CD8^+^ T cells (Figure 2, G and H). These observations indicate that both a reduction in CD8^+^ T cell abundance and a marked increase in their functional exhaustion may critically impair immunity and subsequently contribute to organ dysfunction during sepsis.

### Adoptive transfer of CD8^+^ T cells reduces septic mortality by preserving organ function.

**Figure 3:**
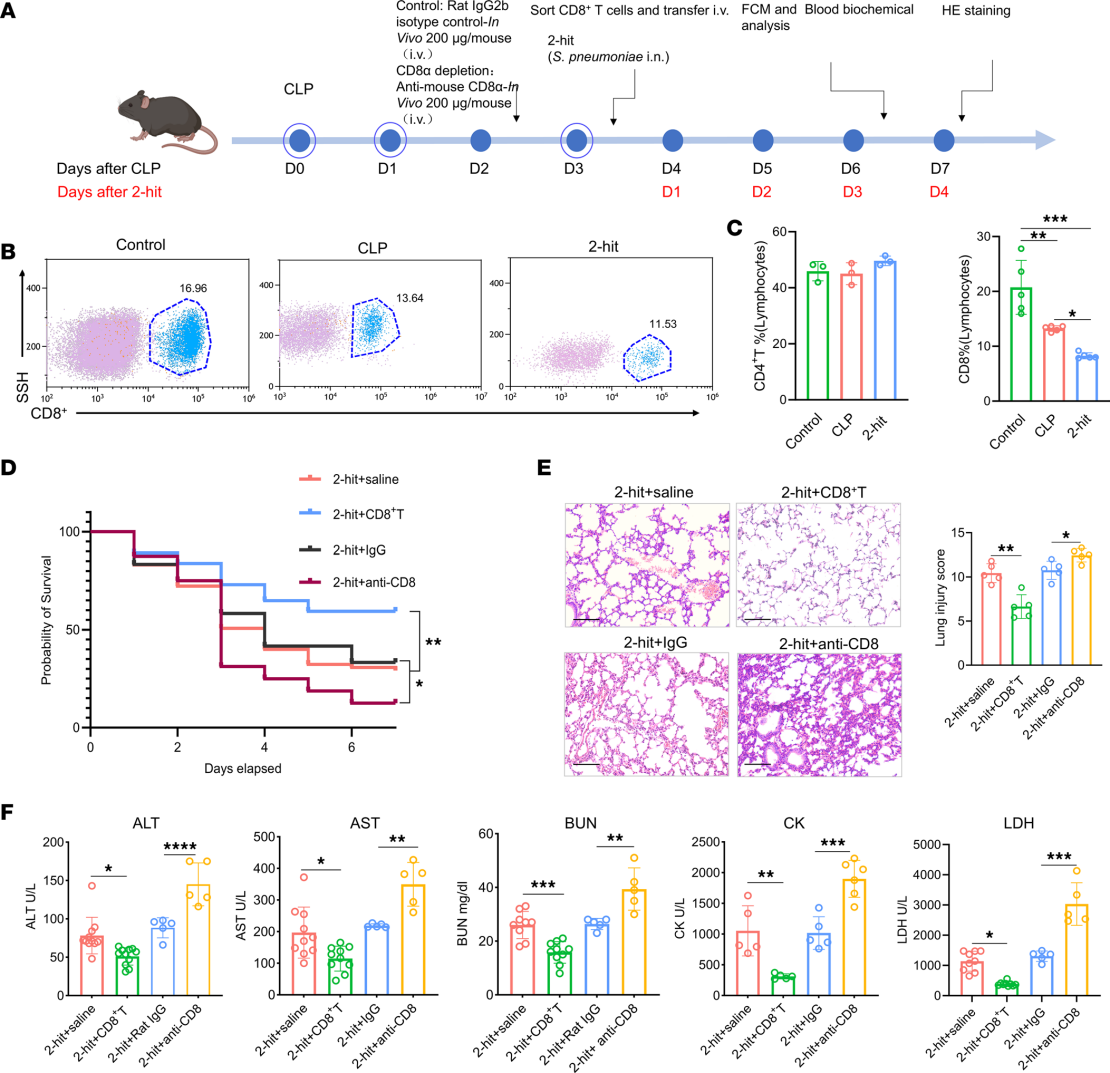
Adoptive transfer of CD8^+^ T cells reduces the sepsis mortality rate by preserving organ function. (**A**) Schematic of the animal experimental procedure. Mice were subjected to CLP followed by intranasal challenge with *Streptococcus pneumoniae* (Sp) as the second hit (2-hit). (**B**) Representative flow cytometry plots of CD8^+^ T cells from the peripheral blood of control, CLP-induced sepsis model, and 2-hit model mice. (**C**) Comparison of CD4^+^ and CD8^+^ T cell proportions in the peripheral blood of healthy control, CLP-induced sepsis model, and 2-hit model mice. (**D**) Kaplan-Meier survival analysis of septic mice following 2-hit model and/or therapeutic administration of CD8^+^ T cells (5 × 10^5^ per mouse) or 0.9% saline (*n* = 63 for saline, *n* = 37 for CD8^+^ T cell administration, *n* = 32 for anti-CD8 antibody administration, *n* = 24 for isotype antibody administration). (**E**) Representative H&E-stained lung sections and corresponding histological injury scores (*n* = 5, original magnification, ×200; scale bar: 100 μm). (**F**) Serum enzyme activity in the liver, kidney, and heart of 2-hit mice from the CD8^+^ T cell transfer group, IgG control group, and anti-CD8 group. Data are presented as mean ± SD. Statistical significance was determined using 1-way ANOVA followed by Tukey’s post hoc test (**C**, **E**, and **F**) or log-rank test (**D**). *P < 0.05, **P < 0.01, ***P < 0.001.

Owing to the wide use of multiple organ support therapy, such as continuous blood purification and extracorporeal membrane oxygenation in the intensive care unit (ICU), most patients with sepsis can survive until the initiation of an adaptive immune response but succumb to secondary infections after discharge (24). The CLP model of sepsis is a first-hit mouse model, with most animals dying during the proinflammatory response phase. The second-hit model of sepsis better reflects susceptibility to secondary infection and delayed immunosuppression (25). T lymphocytes play crucial roles in sepsis-induced immunosuppression. In our previous study, we elucidated that the exhaustion of CD4^+^ T cells mediated by TIM3 is closely related to sepsis mortality (6). Here, we analyzed the number of CD4^+^ T cells and CD8^+^ T cells in the peripheral blood of septic second-hit model mice, which can better reflect the late-onset immunosuppression of CLP-induced sepsis model mice (Figure 3A). The results revealed that in the second-hit model mice, there was no significant change in the number of CD4^+^ T cells in the peripheral blood, whereas the proportions of both CD8^+^ T cells and lymphocytes in the peripheral blood significantly decreased in both the CLP model and the second-hit model mice (Figure 3, B and C). We examined the effect of adoptive transfer or antibody-mediated depletion of CD4^+^ T cells and CD8^+^ T cells on the survival rate of sepsis model mice (depletion efficiency confirmed in Supplemental Figure 3). The results revealed that CD4^+^ T cell transfer did not reduce the mortality rate, whereas CD8^+^ T cell transfer significantly reduced the mortality rate (*P* = 0.0223, Figure 3D and Supplemental Figure 2A). CD8^+^ T cell transfer also reduced the severity of septic lung damage in second-hit mice. The alveolar septum thickening, alveolar congestion, leukocyte infiltration, and edema observed in the lungs of second-hit CD8^+^ T cell recipient mice via H&E staining were significantly alleviated (*P* = 0.0011 for the lung pathology score, Figure 3E Supplemental Figure 2C). Enzyme levels in the heart, liver, and kidney also revealed the protective effect of CD8^+^ T cell transfer during sepsis-induced immunosuppression. The levels of metabolic indicators in the heart (creatine kinase, CK; lactate dehydrogenase, LDH), liver (aspartate transaminase, AST; alanine transaminase, ALT), and kidney (blood urea nitrogen, BUN) in the CD8^+^ T cell adoptive group were significantly lower than those in the control group (*P* = 0.0475 for ALT, *P* = 0.0137 for AST, *P* = 0.0089 for CK, *P* = 0.0329 for LDH, and *P* = 0.0268 for BUN; Figure 3F). Taken together, these results suggest that the proportion of CD8^+^ T cells in peripheral blood significantly decreases during septic immunosuppression and that the adoptive transfer of CD8^+^ T cells decreases the sepsis mortality rate by preserving organ function.

### The expression of HDAC1 on CD8^+^ T cells is increased in patients with sepsis.

**Figure 4:**
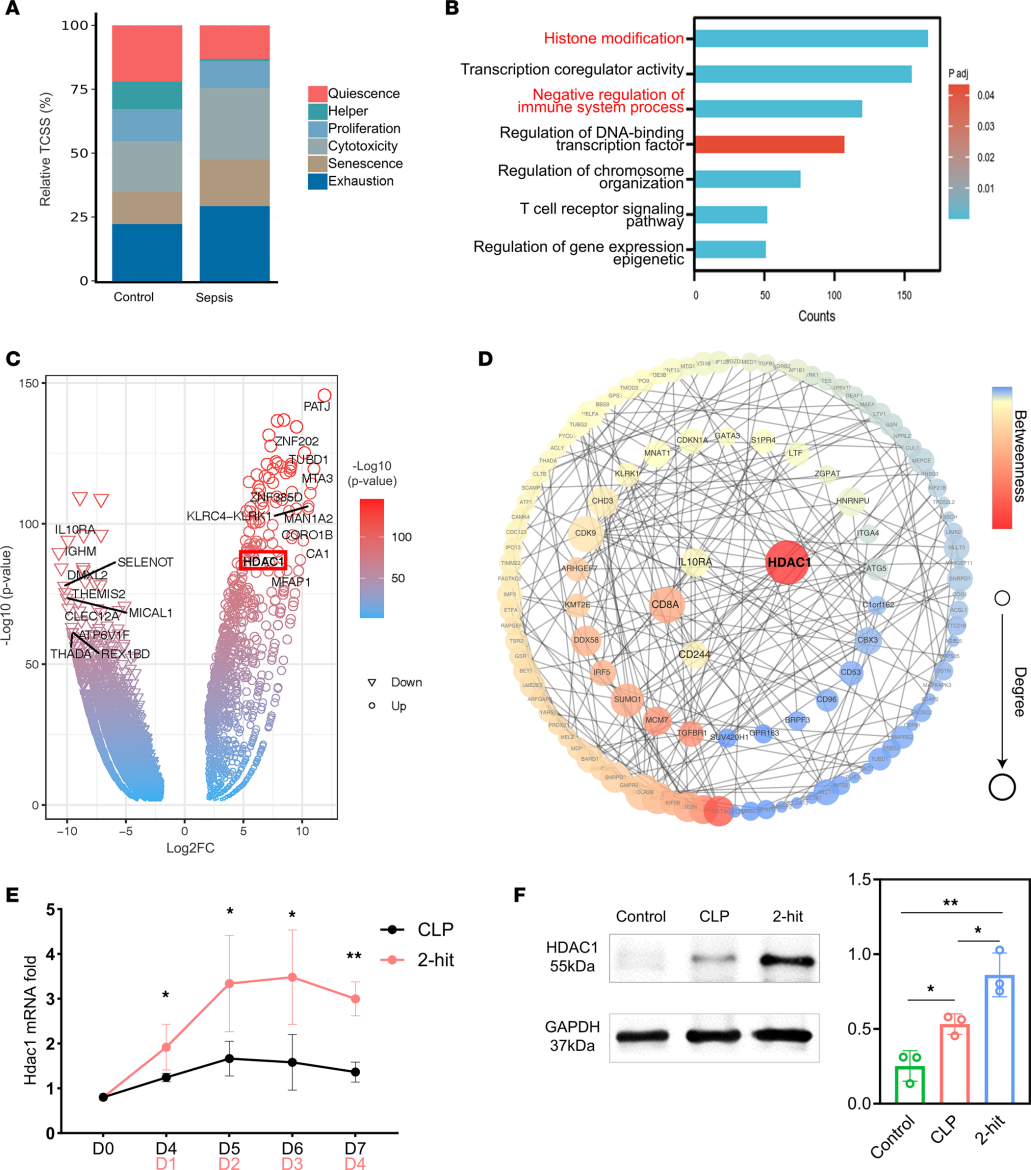
The expression of *HDAC1* in CD8^+^ T cells from patients with sepsis-induced immunosuppression. (**A**) Proportions of different functional CD8^+^ T cell subsets in healthy volunteers and patients with sepsis estimated using the TCellSI package based on RNA-Seq data. (**B**) Gene Ontology (GO) enrichment analysis of differentially expressed genes in CD8^+^ T cells from patients with sepsis versus healthy volunteers. (**C**) Volcano plot depicting differentially expressed genes in CD8^+^ T cells from patients with sepsis compared with those from healthy volunteers. (**D**) Protein-protein interaction network analysis of differentially expressed genes using the STRING database. (**E**) Quantitative PCR analysis of *Hdac1* mRNA levels in the spleens of CLP mice and 2-hit model mice at different time points. (**F**) Western blot analysis of HDAC1 protein expression in the spleen. **E** and **F** are presented as mean ± SD. One-way ANOVA. *P < 0.05, **P < 0.01, ***P < 0.001.

Immunosuppression often occurs 7 to 10 days after the onset of sepsis (1, 7). We collected peripheral blood from 20 healthy volunteers and 20 patients with sepsis 14 days after sepsis diagnosis (the clinical characteristics of the patients with sepsis are shown in Supplemental Table 1). RNA-Seq was used to analyze the gene expression of CD8^+^ T cells. Comparative analysis revealed a significant increase in the proportion of exhausted CD8^+^ T cells in patients with sepsis, accompanied by the upregulation of numerous genes involved in epigenetic modifications and immunosuppression, as quantified using established exhaustion marker expression profiles (Figure 4, A–C). To identify key regulatory molecules, we conducted STRING protein interaction network analysis on the top 100 differentially expressed genes. By integrating the CytoNCA and cytoHubba algorithms, we identified histone deacetylase 1 (*HDAC1*) as a critical hub gene that was significantly upregulated (6.07-fold increase, *P* < 0.0001), with an enrichment score of 65 and the highest betweenness centrality value (Figure 4D). Gene Ontology (GO) pathway analysis revealed that the differentially expressed genes were significantly associated with pathways related to histone modification, chromatin structure, transcription factor regulation, and negative regulation of immune responses (Figure 4B).

HDAC1, a class I histone deacetylase, has emerged as a key regulator in various biological processes. By mediating histone deacetylation, HDAC1 induces chromatin compaction, thereby silencing gene transcription. In sepsis, HDAC1 may modulate immune responses by regulating the expression of inflammatory genes, potentially exacerbating immunosuppression. Therefore, we further investigated the role of HDAC1 in sepsis. Therefore, we measured the transcript level of HDAC1 in CD8^+^ T cells from septic mice. The results revealed that the expression of the HDAC1 gene increased with the progression of sepsis-induced immunosuppression (Figure 4E). We also observed the protein expression of HDAC1. The Western blot results revealed that HDAC1 expression significantly increased after CLP and second-hit in CD8^+^ T cells (*P* = 0.047 and *P* = 0.024, Figure 4F).

To verify the specificity of HDAC1, we comprehensively assessed the expression of other HDAC family members (HDAC1–11). In human bulk RNA-Seq data, while both *HDAC1* and *HDAC2* were upregulated in septic CD8^+^ T cells, other isoforms (*HDAC3–9*) were downregulated (Supplemental Figure 4A). However, distinct dynamics were observed in the murine model. Single-cell analysis revealed that *Hdac1* expression progressively increased with disease progression, whereas *Hdac2*, *Hdac4*, *Hdac7*, and *Hdac8* expression levels decreased, and other isoforms remained barely detectable in T cells (Supplemental Figure 4B). Validatory qPCR further confirmed that *Hdac1* was the sole family member significantly upregulated in the severe second-hit group compared with the CLP group (Supplemental Figure 4C). These findings identify HDAC1 as the dominant epigenetic regulator specifically associated with the progression of sepsis-induced T cell exhaustion, justifying our further investigation into its role in sepsis-induced immunosuppression.

### Pharmacological inhibition of HDAC1 reduces the mortality rate of sepsis model mice by inhibiting CD8^+^ T cell exhaustion.

**Figure 5:**
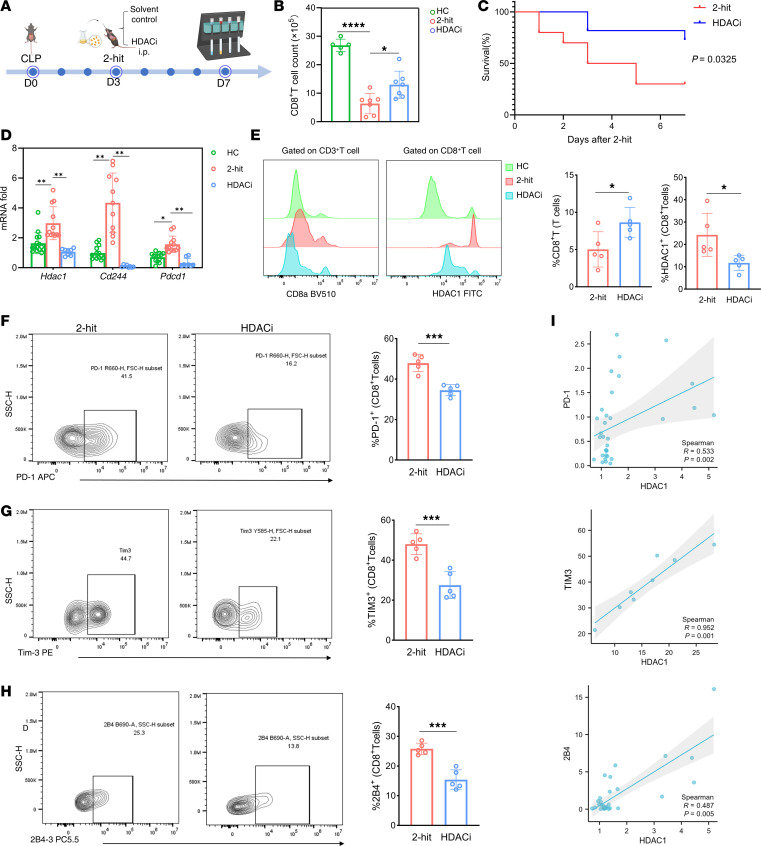
HDAC1 inhibition alleviates CD8^+^ T cell exhaustion and increases the survival rate of mice. (**A**) Schematic timeline of the experimental design. The mice underwent CLP (day 0) followed by intranasal administration of *Streptococcus pneumoniae* as a second hit (day 3). HDAC inhibitors (HDACis) were i.p. administered after the second hit, and the analyses were performed on day 7. (**B**) Quantification of CD8^+^ T cell counts in the peripheral blood of healthy, 2-hit model, and HDACi-treated mice. (**C**) Kaplan-Meier survival analysis of 2-hit model mice treated with HDACis (*n* = 25 per group, *P =* 0.0325). (**D**) Relative mRNA expression levels of *Hdac1*, *Cd244*, and *Pdcd1* in splenic CD8^+^ T cells from CLP-induced sepsis model, 2-hit model, and HDACi-treated mice. (**E**) Representative flow cytometry histograms showing CD8 and HDAC1 expression in T cells. The quantification of the percentages of CD8^+^ T cells and HDAC1^+^ CD8^+^ T cells is shown. (**F**–**H**) Flow cytometry plots and quantification of the exhaustion markers PD-1 (**F**), TIM3 (**G**), and 2B4 (**H**) on CD8^+^ T cells from 2-hit model and HDACi-treated mice. (**I**) Correlation analysis between HDAC1 expression and the exhaustion markers PD-1, TIM3, and 2B4 on CD8^+^ T cells. Spearman’s correlation coefficients (*R*) and *P* values are shown. Data are presented as mean ± SEM. Statistical significance was determined by 1-way ANOVA with Tukey’s multiple-comparison test (**B** and **D**), unpaired 2-tailed Student’s *t*-test (**E**–**H**), log-rank test (**C**), or Spearman’s correlation (**I**). *P < 0.05, **P < 0.01, ***P < 0.001, ****P < 0.0001.

As cytotoxic T lymphocytes, CD8^+^ T cells are crucial for regulating immune responses and the development of inflammation. However, little information is available concerning the role of HDAC1 in CD8^+^ T cells. Therefore, an HDAC1 inhibitor (mocetinostat) was used to block the expression and activity of HDAC1 in CD8^+^ T cells. During acute infection, once pathogens are cleared or inflammation subsides, effector CD8^+^ T cells further differentiate into memory T cells (26). During chronic infection, persistent antigen exposure and sustained inflammatory signaling drive effector T cells into an exhausted state (Tex) (27). The expression of various inhibitory molecules of Tex continues to increase (such as PD-1, 2B4, and TIM3), and key transcription factors (T-bet, Eomes, Blimp-1, and NFAT/AP-1) undergo functional and metabolic alterations. These changes ultimately result in the loss of T cell function and induce cell death, thereby altering the immune status of sepsis and contributing to adverse outcomes (6). Therefore, we investigated the impact of pharmacological inhibition of HDAC1 on the mortality rate of sepsis model mice and the expression of inhibitory costimulatory molecules on CD8^+^ T cells (Figure 5A). We initially investigated whether mocetinostat affects the number of CD8^+^ T cells in the peripheral blood of septic mice. Mocetinostat treatment significantly increased the number of CD8^+^ T cells in the peripheral blood in the sepsis immunosuppression second-hit model mice (Figure 5B) and improved survival during the observation period (*P* = 0.0325, Figure 5C). We next assessed whether HDAC1 inhibition affected the expression of exhaustion-related inhibitory receptors. At the mRNA level, septic mice exhibited markedly increased expression of *Hdac1*, *Pdcd1* (encoding PD-1), and *Cd244* (encoding 2B4), which was significantly reduced by mocetinostat treatment (*P* < 0.001, Figure 5D). The protein levels of inhibitory costimulatory molecules analyzed by FACS was consistent with their transcript levels (*P* = 0.0003 for PD-1, *P* = 0.0006 for TIM3, and *P* = 0.0002 for 2B4; Figure 5, E–H). We speculated that HDAC1-mediated histone deacetylation may be closely related to CD8^+^ T cell exhaustion in sepsis-induced immunosuppression. Further correlation analysis revealed that the expression of HDAC1 was significantly positively correlated with the expression of PD-1, TIM3, and 2B4 (Spearman’s *R =* 0.533*, P =* 0.002; *R =* 0.952*, P =* 0.001; and *R =* 0.487, *P =* 0.005, respectively*;* Figure 5I).

To precisely delineate the stage of exhaustion associated with HDAC1 expression, we first analyzed its distribution across CD8^+^ T cell subsets at the single-cell level. Feature plots and violin plots confirmed that *Hdac1* expression was significantly upregulated in the exhausted CD8^+^ T cell (CD8_Tex) cluster compared with naive and effector memory phenotypes (Supplemental Figure 5, A–C). Building on this observation, we performed a global correlation analysis using raw gene expression counts. As shown in Supplemental Figure 5D, *Hdac1* exhibited a general positive association with exhaustion markers (*Pdcd1*, *Havcr2*, and *Cd244*) across the CD8^+^ T cell population. To mitigate technical dropout effects and resolve these relationships across distinct disease states, we subsequently applied MAGIC imputation. This refined analysis revealed robust, group-consistent positive correlations between *Hdac1* and *Havcr2* (encoding TIM3) across all experimental groups (Supplemental Figure 5, E–G). Notably, *Hdac1* exhibited a stronger and steeper positive correlation with the canonical terminal exhaustion marker *Havcr2* compared with *Pdcd1*. This distinction likely stems from the broader expression of PD-1 on effector T cells, whereas TIM3 is restricted to terminally differentiated subsets (28). These single-cell data corroborate our bulk findings, suggesting that HDAC1 upregulation is specifically linked to the transition toward a terminally exhausted phenotype. Collectively, these results indicate that pharmacological inhibition of HDAC1 might reduce sepsis mortality by preventing CD8^+^ T cell exhaustion.

### Pharmacological inhibition of HDAC1 maintains the activity of CD8^+^ T cells.

**Figure 6:**
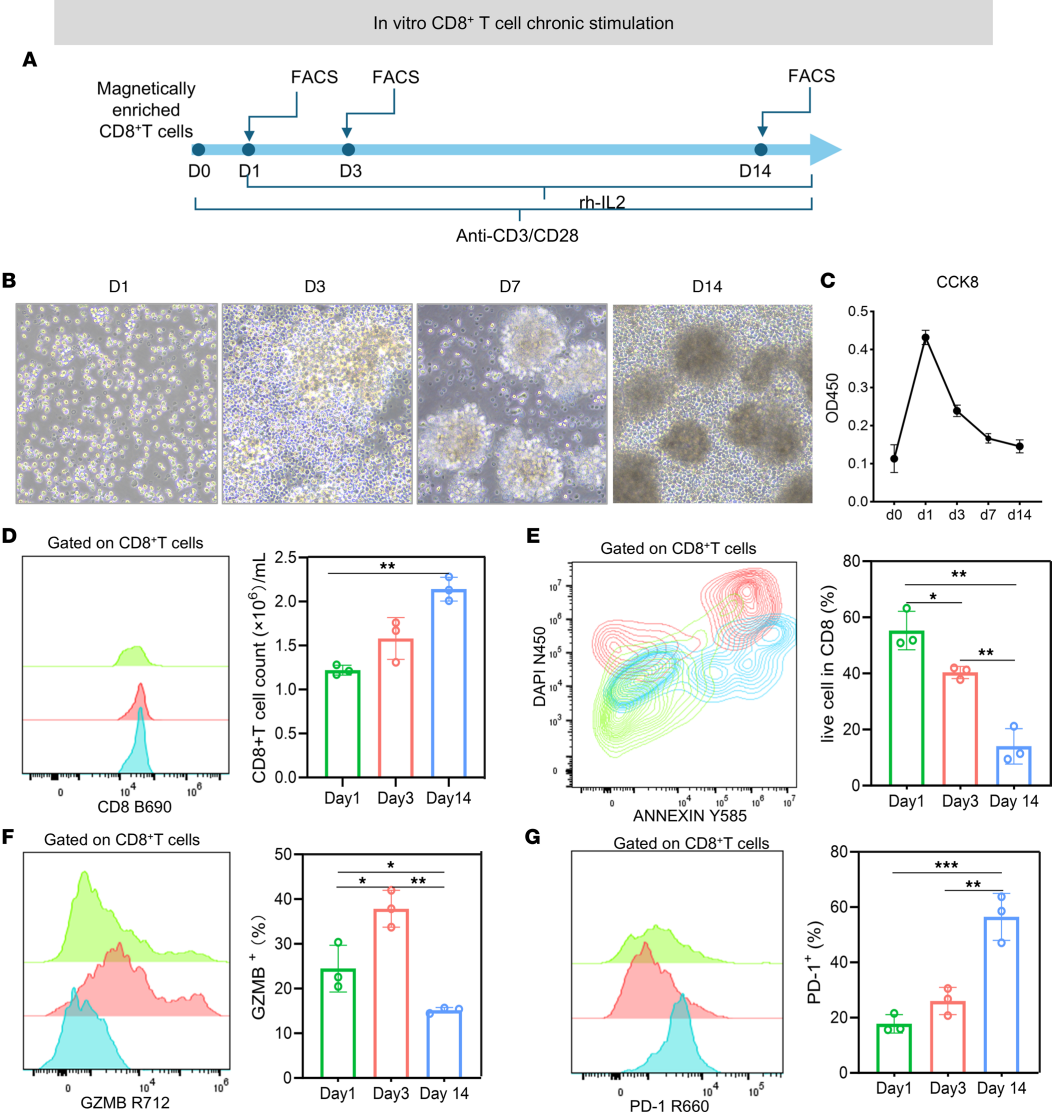
Chronic stimulation induces phenotypic and functional exhaustion in CD8^+^ T cells in vitro. (**A**) Schematic timeline of the in vitro chronic stimulation model. CD8^+^ T cells were stimulated with anti-CD3/CD28 antibodies and rhIL-2 for 14 days. Flow cytometry analyses were performed on days 1, 3, 7, and 14. (**B**) Representative bright-field microscopy images of CD8^+^ T cells on days 1, 3, 7, and 14, showing progressive morphological changes and clustering over time. Scale bar: 100 μm. (**C**) CCK-8 assay showing the viability (OD450) of CD8^+^ T cells at different time points. (**D**) Flow cytometry histograms and quantification of the CD8^+^ T cell count. (**E**) Flow cytometry plots showing live/dead staining (DAPI/Annexin Y585) of CD8^+^ T cells. The quantification of live cell percentages is shown (*n* = 3). (**F**) Flow cytometry histograms and quantification of GZMB expression in CD8^+^ T cells. (**G**) Flow cytometry histograms and quantification of PD-1 expression in CD8^+^ T cells. Data are presented as mean ± SEM. Statistical significance was determined using 1-way ANOVA. *P < 0.05; ***P < 0.001. HC, healthy control; HDACi, histone deacetylase inhibitor.

**Figure 7:**
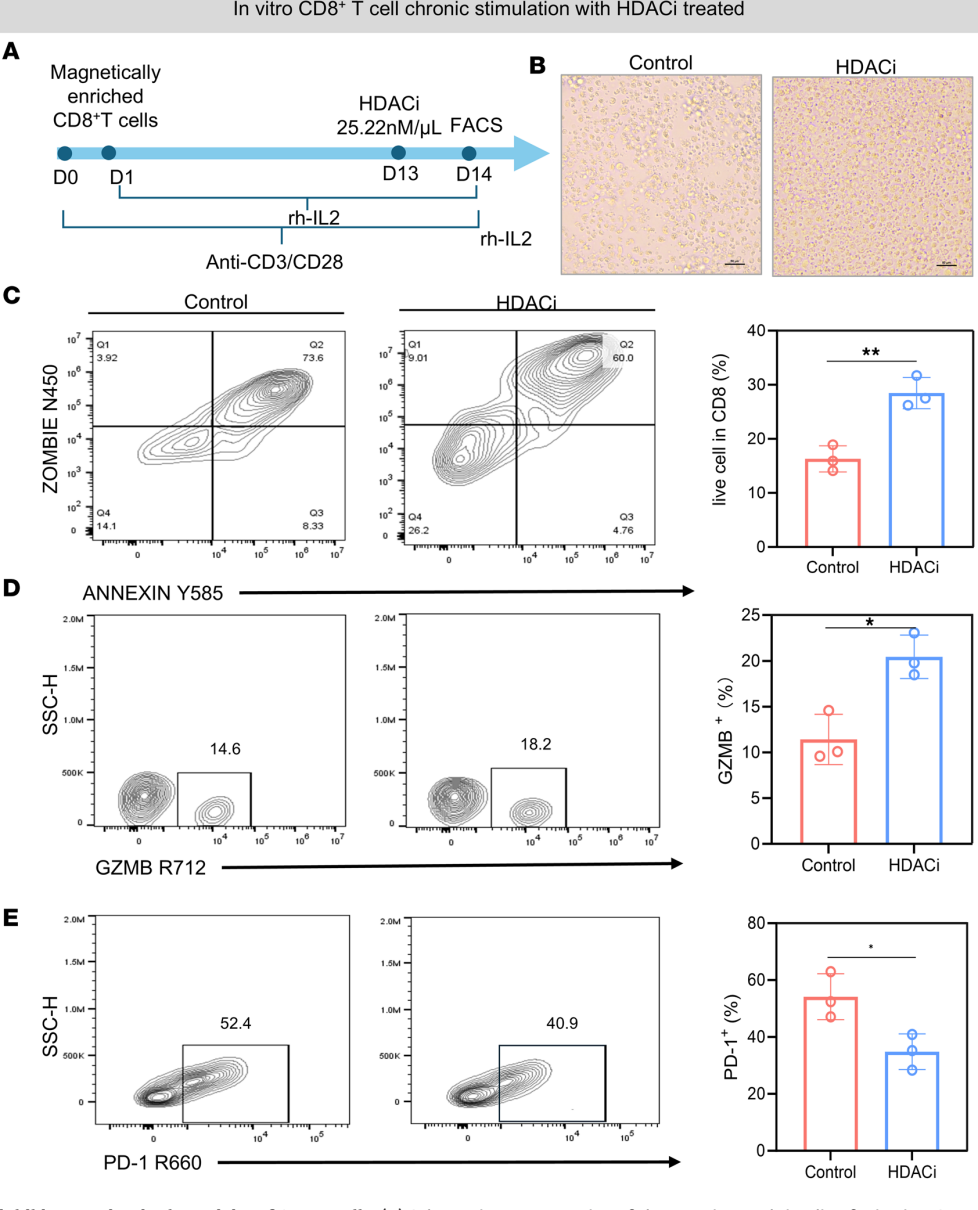
HDAC inhibitors maintain the activity of CD8^+^ T cells. (**A**) Schematic representation of the experimental timeline for in vitro CD8^+^ T cell chronic stimulation. CD8^+^ T cells were stimulated with an anti-CD3/CD28 mAb and rhIL-2 for 14 days. CD8^+^ T cells were treated with an HDACi (0.15 μM) on day 13, followed by flow cytometry analysis on day 14. (**B**) Representative bright-field microscopy images of cultured CD8^+^ T cells. Scale bar: 100 μm. (**C**) Flow cytometry plots showing live/dead cell staining (Zombie N450/Annexin Y585) of CD8^+^ T cells. The quantification of live cell percentages is shown. (**D**) Flow cytometry plots showing GZMB expression in CD8^+^ T cells. (**E**) Flow cytometry plots showing PD-1 expression in CD8^+^ T cells. The data are presented as mean ± SEM. *P < 0.05; **P < 0.01; ***P < 0.001. All flow cytometry analyses were gated on CD8^+^ T cells.

T cell activation is a critical process of the immune response. Over the course of an immune response, T cells may be chronically stimulated, with some proportion becoming exhausted. We applied an in vitro model mimicking CD8^+^ T cell stimulation. T cells were stimulated with recombinant human IL-2 (rhIL-2) and anti-human CD3/CD28 antibodies continuously for 2 weeks (29). This experiment was designed to mimic the physiological activation of T cells. CD3, a component of the T cell receptor complex, delivers the primary activation signal. Moreover, CD28 provides the essential costimulatory signal needed for complete T cell activation. The addition of rhIL-2 further promoted T cell proliferation and survival, replicating the cytokine environment present during sustained immune responses. Therefore, CD8^+^ T cells initially undergo activation and proliferation. However, sustained or prolonged stimulation can lead to exhaustion. Under normal culture conditions, the activity of CD8^+^ T cells gradually decreased over the course of 14 days in vitro (Figure 6, A–C). Granzyme B (GZMB), a cytotoxic protease secreted by CD8^+^ T cells, is an important protease in the immune defense of CD8^+^ T cells. During the exhaustion process of CD8^+^ T cells, the secretion of GZMB by CD8^+^ T cells gradually decreased, whereas the expression of the inhibitory molecule PD-1 gradually increased (Figure 6, D–G). To observe the effects of mocetinostat treatment on the activity and exhaustion of CD8^+^ T cells in vitro, the cells were analyzed by flow cytometry at day 14 (Figure 7A). The proportion of live cells in the mocetinostat treatment group was greater than that in the control group (*P* = 0.005; Figure 7B). The secretion of GZMB by CD8^+^ T cells increased, whereas the expression of the inhibitory molecule PD-1 decreased in the mocetinostat treatment group (*P* = 0.0126 and *P* = 0.0308; Figure 7, C–E). Taken together, these data demonstrate that mocetinostat maintains the activity of CD8^+^ T cells and reduces the expression of negative costimulatory molecules in vitro.

### HDAC1 regulates CD8^+^ T cell exhaustion through modulation of the AP-1/NFAT signaling axis.

**Figure 8:**
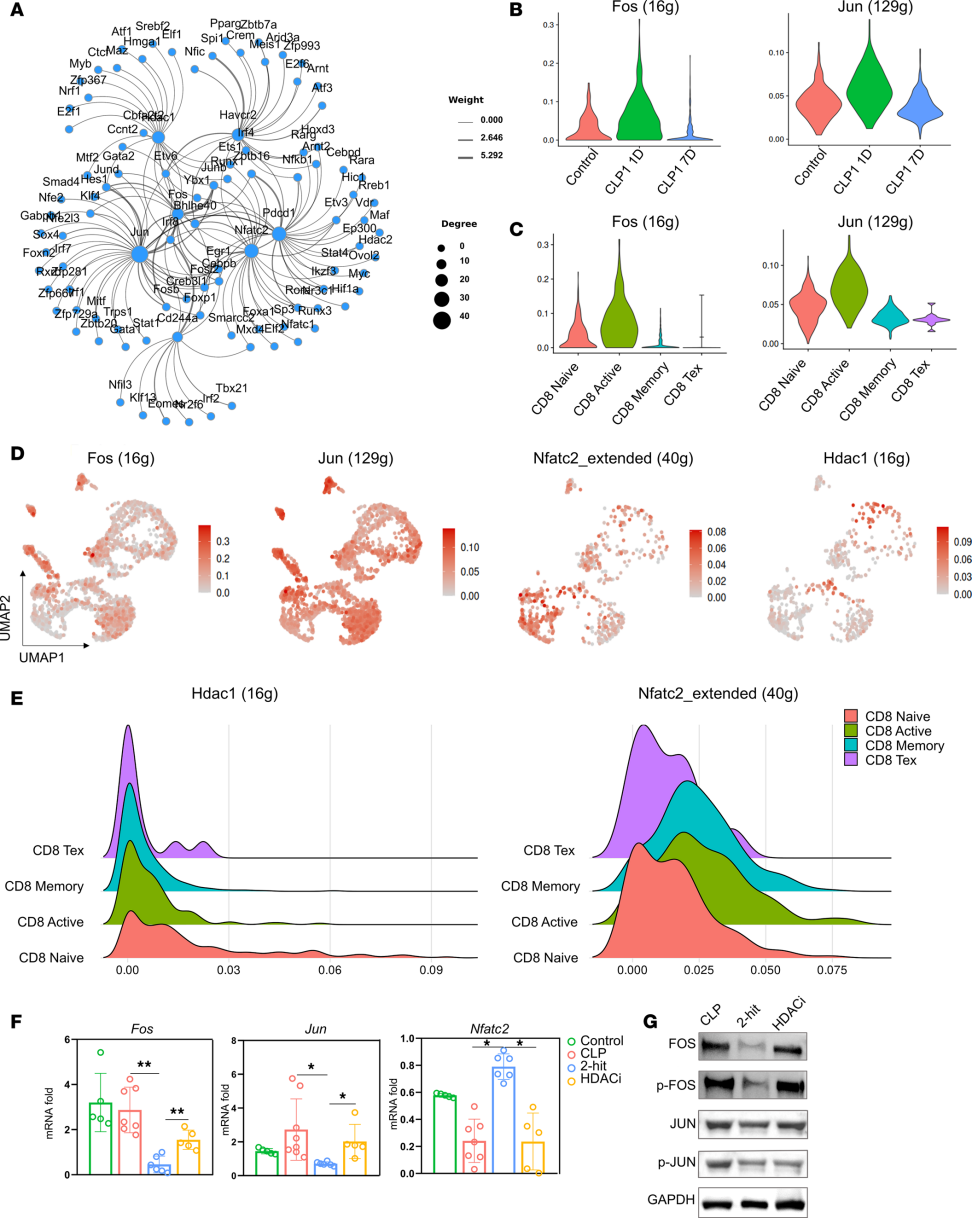
HDAC1 regulates CD8^+^ T cell exhaustion through the modulation of AP-1/NFAT. (**A**) Transcription factor regulatory network predicted, using gene regulatory network (GRN) analysis of RNA-Seq data from septic mice compared with those from healthy controls, highlighting the interactions between HDAC1, AP-1 components, and NFAT1. Node size represents degree centrality; edge thickness indicates the interaction confidence score. (**B**) Violin plots showing the regulon activities (AUC scores) of AP-1 family members (Fos, Jun) in CD8^+^ T cells across healthy controls and septic mice at different time points (CLP day 1, CLP day 7). (**C**) Violin plots quantifying AP-1 regulon activities across distinct CD8^+^ T cell subsets. (**D**) Feature plots displaying the regulon activities (AUC scores) of *Hdac1* and *Nfatc2*, as well as AP-1 family members (*Fos*, *Jun*) on the UMAP projection. (**E**) Ridge plots showing the density distribution of *Hdac1* and *Nfatc2* regulon activity scores across CD8^+^ T cell subsets. (**F**) Relative mRNA expression levels of *Fos*, *Jun*, and *Nfatc2* in splenic CD8^+^ T cells from control, CLP, and 2-hit model mice analyzed by qPCR. (**G**) Representative Western blot showing the protein levels of FOS, JUN, phosphorylated FOS (p-FOS), and phosphorylated JUN (p-JUN), with GAPDH used as a loading control across the experimental groups. Data are presented as mean ± SD. Statistical significance was determined by Wilcoxon test (**B** and **C**) or 1-way ANOVA followed by Tukey’s post hoc test (**F**). *P < 0.05, **P < 0.01, ***P < 0.001, ****P < 0.0001.

To elucidate the molecular mechanisms underlying HDAC1-mediated CD8^+^ T cell exhaustion in sepsis, we examined the transcriptional networks regulating T cell activation and exhaustion. During physiological T cell activation, MHC-antigen complexes engage the T cell receptor, initiating signaling cascades that activate AP-1, a heterodimeric transcription complex comprising JUN and FOS proteins. AP-1 typically forms a functional complex with dephosphorylated NFAT to drive the transcription of costimulatory genes. Conversely, in the absence of AP-1, NFAT independently increases the expression of inhibitory costimulatory molecules (30). To investigate this mechanism in sepsis, we performed single-cell transcriptome sequencing combined with SCENIC transcription factor analysis. Our results revealed that the regulon activities (AUC scores) of key AP-1 components, *Fos* and *Jun*, exhibited a progressive decline throughout the course of sepsis and were virtually abolished in the exhausted subset compared with naive and effector memory cells, and these factors were predicted to be direct targets of *Hdac1* (Figure 8, A–C). Conversely, the regulon activity of *Hdac1* was sustained in these cells and accompanied by elevated activity of *Nfatc2* (encoding NFAT1) (Figure 8, D and E). This specific expression pattern, characterized by the retention of HDAC1 and NFAT1 activity alongside a marked reduction in AP-1 signaling, provides in vivo single-cell evidence supporting a model where HDAC1 disrupts the AP-1/NFAT transcriptional balance. Further validation in our animal models confirmed that the expression of Jun and Fos, components of the AP-1 complex, was markedly decreased in CD8^+^ T cells from second-hit model septic mice. Importantly, treatment with an HDAC1 inhibitor restored their expression to near-normal levels (Figure 8, F and G). These findings suggest that during sepsis, HDAC1 may promote CD8^+^ T cell exhaustion by downregulating AP-1 expression, thereby disrupting the AP-1/NFAT balance. This imbalance appears to favor NFAT-mediated induction of inhibitory genes, including PD-1 and other exhaustion markers.

**Figure 9:**
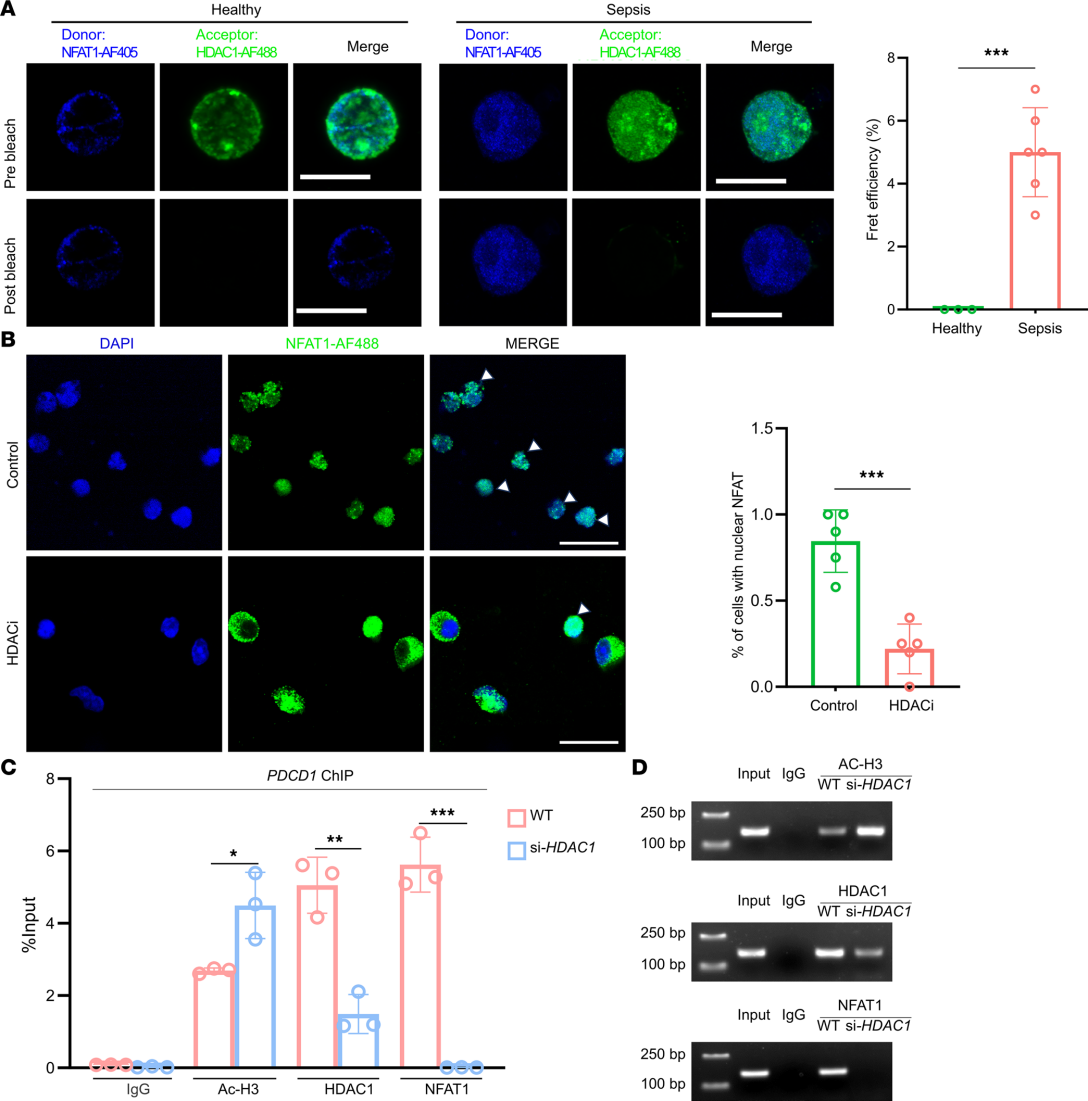
HDAC1 interacts with NFAT1 to promote its nuclear localization and transcriptional regulation of *Pdcd1*. (**A**) Fluorescence resonance energy transfer (FRET) analysis of the HDAC1-NFAT1 interaction in CD8^+^ T cells from healthy volunteers and patients with sepsis. Representative images showing donor (NFAT1-AF405) and acceptor (HDAC1-AF488) samples before and after photobleaching. Quantification of FRET efficiency. Scale bars: 10 μm. (**B**) Immunofluorescence images showing the nuclear localization of NFAT1 in CD8^+^ T cells from the control or HDACi-treated groups. Quantification revealed a significant reduction in nuclear NFAT1 localization following HDACi treatment. Scale bar: 20 μm. (**C**) ChIP–qPCR analysis showing the percentages of HDAC1, acetylated histone H3 (Ac-H3), and NFAT1 bound to the *PDCD1* promoter in WT and HDAC1 siRNA-transfected Jurkat cells. ChIP with IgG served as a negative control. (**D**) Agarose gel electrophoresis visualization of ChIP–qPCR products showing that HDAC1, Ac-H3, and NFAT1 bind to the *PDCD1* promoter in WT and *HDAC1* siRNA-transfected cells. Input DNA and IgG immunoprecipitates served as positive and negative controls, respectively. Data are presented as mean ± SEM. Statistical significance was determined using 1-way ANOVA followed by Tukey’s post hoc test. *P < 0.05, **P < 0.01, ***P < 0.001.

Recent studies have expanded our understanding of HDAC functions beyond their classical role in transcriptional repression. For example, HDAC3, another class I HDAC family member, also has transcriptional activity and plays dual roles in LPS-induced inflammatory responses (31). Therefore, we investigated whether HDAC1 has transcriptional activation independent of its histone deacetylase activity to upregulate inhibitory immune checkpoint molecules that induce CD8^+^ T cell exhaustion. Fluorescence resonance energy transfer (FRET) analysis was used for direct molecular interaction analysis between HDAC1 and NFAT1. Compared with CD8^+^ T cells from healthy controls, those from patients with sepsis exhibited significantly greater FRET efficiency between HDAC1 and NFAT1 (*P* < 0.001; Figure 9A), indicating increased NFAT1 and HDAC proximity and potential protein-protein interactions during sepsis. This observation suggests that in addition to its canonical histone deacetylase function, HDAC1 may directly interact with transcription factors to modulate gene expression. Furthermore, immunofluorescence experiments confirmed that treatment with HDACis significantly reduced NFAT1 nuclear translocation in exhausted CD8^+^ T cells (Figure 9B), providing additional evidence for the role of HDAC1 in regulating NFAT1 nuclear localization and subsequent transcriptional activity.

To further clarify the mechanism by which HDAC1 regulates *PDCD1* (encoding PD-1) expression via NFAT1, we performed ChIP assays on the *PDCD1* promoter region. We first established an in vitro model using PMA/ionomycin-stimulated Jurkat T cells, which demonstrated significant increases in CD8 expression and exhaustion markers after stimulation (Supplemental Figure 6, A–C). We generated HDAC1-knockdown Jurkat T cells using 3 specific siRNAs and achieved 85% to 95% HDAC1 knockdown, as confirmed using Western blot (Figure S6D) and flow cytometry analyses (Supplemental Figure 6E). ChIP-qPCR analysis revealed significant HDAC1 occupancy at the *PDCD1* promoter in WT cells compared with HDAC1-knockdown cells (5.1% vs. 1.5% input, *P* = 0.0028). Consistent with the canonical function of HDAC1, acetylated histone H3 levels at the *PDCD1* promoter were significantly lower in WT cells than in HDAC1-knockdown cells (2.8% vs. 4.5% input, *P* = 0.0275). Importantly, NFAT1 occupancy at the *PDCD1* promoter was robust in the WT cells (5.5% input) but virtually undetectable in the knockdown cells (*P* = 0.0002), and visualization of the ChIP-qPCR products by agarose gel electrophoresis further confirmed these findings (Figure 9, C and D). These findings demonstrate that HDAC1 and NFAT1 co-occupy the PD-1 promoter, supporting a direct regulatory role for HDAC1 in facilitating NFAT1-mediated PD-1 transcription during T cell exhaustion. Collectively, our data reveal a dual role of HDAC1 in driving CD8^+^ T cell exhaustion: it downregulates AP-1 components (Jun and Fos), promotes the transcription of inhibitory costimulatory molecules, and facilitates NFAT1 nuclear translocation to promote the transcription of inhibitory costimulatory molecules.

## Discussion

Sepsis remains a leading cause of mortality in ICUs, with sepsis-induced immunosuppression emerging as a critical pathophysiological process contributing to secondary infections and increased mortality after discharge (32, 33). Our study highlights the pivotal role of CD8^+^ T cells in sepsis-induced immunosuppression. We demonstrated that the proportion of CD8^+^ T cells in the peripheral blood significantly decreased during sepsis, which was correlated with increased disease severity and mortality. Importantly, adoptive transfer of CD8^+^ T cells effectively reduced the mortality rate of sepsis model mice by preserving organ function, suggesting a potential therapeutic strategy for sepsis-induced immunosuppression. Interestingly, we observed that CD8^+^ T cells exerted a significant mediating effect on clinical outcomes, whereas CD4^+^ T cells did not. One possible explanation is related to the timing of blood sampling in our study, which was performed on day 7 after ICU admission. Previous studies have shown that CD4^+^ T cells undergo early depletion in the acute phase of sepsis and may partially recover during the later phase — though this recovery may be numerical rather than functional (34, 35) — whereas CD8^+^ T cells exhibit persistent dysfunction and exhaustion characteristics that are more pronounced during prolonged immunosuppression. This temporal difference in immune dynamics could explain why CD8^+^ T cells retained a stronger association with disease severity at this time point.

Furthermore, we analyzed the expression profile of CD8^+^ T cells in the peripheral blood of patients with sepsis and found that HDAC1 was significantly upregulated in CD8^+^ T cells from patients with sepsis. HDAC1, a class I histone deacetylase, is known to mediate histone deacetylation, leading to chromatin modification and transcriptional silencing. HDACs are a family of enzymes that deacetylate lysine residues in both histone and nonhistone proteins. Deacetylation of histone lysine residues results in a more condensed chromatin structure, thereby reducing DNA accessibility and repressing gene transcription (36). To date, 18 HDAC isoenzymes have been identified and classified into 4 categories on the basis of their structural homology (37). Class I HDACs, which include the zinc-dependent enzymes HDAC1, 2, 3, and 8, play crucial roles in regulating gene expression, cell survival, and proliferation (38). Given their importance, small-molecule inhibitors targeting class I HDACs are considered potential therapeutic agents for various diseases, including cancer (39), inflammatory diseases (40), cardiac and pulmonary diseases (41), and neurological disorders (42).

In the context of sepsis, previous studies have predominantly focused on the therapeutic potential of broad-spectrum HDACis. Agents such as butyrate and valproic acid, which act as pan-HDACis targeting both class I and class IIa isoenzymes, improve survival primarily by mitigating innate immune dysregulation, including macrophage-mediated inflammation and autophagy (18, 19). Investigations into specific isoforms have similarly concentrated on the innate compartment. For instance, HDAC3 has been identified as a regulator of macrophage inflammatory responses via the NF-κB pathway (30), and HDAC6 is known to modulate endothelial permeability and cytokine production during sepsis-induced lung injury (43). Similarly, class I members like HDAC2 have been recently implicated in enhancing the antimicrobial activity of neutrophils by promoting the formation of neutrophil extracellular traps (44). However, these approaches often overlook the adaptive immune response or utilize nonselective inhibitors with potential for widespread off-target pleiotropic effects.

In contrast, our study provides a distinct perspective by focusing on the adaptive immune response. We identified HDAC1 rather than other family members as the dominant epigenetic driver of T cell exhaustion. Guided by this specific target, we selected mocetinostat, a potent inhibitor with high selectivity for class I HDACs (particularly HDAC1), to precisely intervene in this pathway. Consistent with this targeted approach, we observed that pharmacological inhibition of HDAC1 maintained the activity of CD8^+^ T cells in vitro; furthermore, it reduced the mortality of mice with sepsis-induced immunosuppression by blocking CD8^+^ T cell exhaustion. Therefore, we show that HDAC1 contributes to CD8^+^ T cell exhaustion by modulating the expression of inhibitory costimulatory molecules such as PD-1 and TIM3. To elucidate the mechanisms involved, we examined the transcriptional networks regulating T cell activation and exhaustion. Our scRNA-Seq and transcription factor analysis revealed that HDAC1 downregulated the expression of AP-1 components (JUN and FOS), which are essential for T cell activation. This downregulation disrupts the balance between AP-1 and NFAT, promoting NFAT-mediated transcription of inhibitory genes. Additionally, we found that HDAC1 directly interacted with NFAT1, promoting its nuclear translocation and further increasing the expression of inhibitory costimulatory molecules. This dual role of HDAC1 in modulating transcription factors and directly interacting with NFAT1 highlights its importance in driving CD8^+^ T cell exhaustion during sepsis.

Our findings identify HDAC1 inhibition as a promising therapeutic strategy for reversing T cell exhaustion, but repurposing mocetinostat for sepsis warrants careful consideration. Although mocetinostat is currently evaluated in oncology with established protocols for monitoring adverse events such as myelosuppression or cardiac effects (45, 46), patients with sepsis present unique hemodynamic fragility that demands rigorous safety scrutiny. However, the therapeutic context in sepsis differs fundamentally from oncology. Unlike the chronic, continuous dosing required for tumor suppression, reversing immunoparalysis is envisioned here as a short-term, “pulse” administration aimed specifically at halting the progression of T cell exhaustion. Crucially, preclinical evidence from non-oncology models supports the safety of this approach. A recent study demonstrated that mocetinostat (2–10 mg/kg) effectively protected against osteoarthritis in mice without causing apparent systemic adverse reactions or distress (47). Furthermore, in a rat model of congestive heart failure, mocetinostat treatment (20 mg/kg/day) was shown to actually improve cardiac function and reduce fibrosis without systemic toxicity (48). Given that our study utilizes comparable doses (10 mg/kg), and considering the proposed short-term dosing regimen, we postulate that the risk of exacerbating hemodynamic compromise in patients with sepsis is manageable. Nevertheless, future translational efforts must prioritize phase I dose-escalation studies to identify a therapeutic window that restores immune competence without exacerbating metabolic or hemodynamic compromise in fragile septic hosts.

Our study focused on pneumonia-associated sepsis because it remains the leading cause of sepsis-related mortality and frequently leads to immunosuppression. In this specific model, pulmonary inflammation and epithelial barrier disruption creates a microenvironment that favors HDAC1 upregulation and CD8^+^ T cell exhaustion. However, given the heterogeneity of sepsis, where the infection site and pathogen dictate distinct immune trajectories, caution is warranted in generalizing our findings to other etiologies such as abdominal or urinary sepsis in which polymicrobial infections, systemic endotoxin exposure, and unique cytokine profiles may drive alternative exhaustion pathways. Despite these upstream variations, T cell exhaustion emerges as a convergent state of immunosuppression observed in the chronic phase of sepsis survivors across diverse etiologies. This shared phenotype highlights exhaustion as a common therapeutic target, even if the molecular drivers differ. Future work should systematically compare HDAC1 activity and related pathways across different sepsis models and patient cohorts to determine whether HDAC1 functions as a universal brake or a pneumonia-specific checkpoint in sepsis immunosuppression.

In conclusion, our study underscores the critical role of HDAC1 in driving CD8^+^ T cell exhaustion during sepsis and highlights its potential role in therapeutic treatment. By elucidating the molecular mechanisms underlying HDAC1-mediated immunosuppression, we provide a foundation for developing immunomodulatory therapies for sepsis treatment.

## Methods

### Sex as a biological variable.

Sex was not considered as a biological variable in this study. Human samples included both male and female donors. Mouse experiments used male and female mice.

### Study population.

In the MIMIC-IV database, a total of 247,366 individuals were recorded to be admitted to the hospital, of which 76,540 were admitted to the ICU. Hospitalization information for patients with sepsis was extracted using the International Classification of Diseases, Ninth Revision (ICD-9) codes (995.91, 995.92, 785.52) and the Tenth Revision (ICD-10) codes (R6.20–R65.21), resulting in the inclusion of 18,220 patients, of whom 6,596 had the complication of pneumonia defined by corresponding respiratory infection codes. After further screening, patients meeting the following criteria were excluded: (a) were younger than 18 years at the time of first admission; (b) had malignant tumors; and (c) had autoimmune diseases. Ultimately, 2,467 patients were included in this study. Ethical access was obtained through Collaborative Institutional Training Initiative (CITI) certification, enabling extraction of clinically relevant variables for analysis.

### Patient samples.

Peripheral blood samples from 140 patients with sepsis and 20 healthy controls were obtained from Daping Hospital of Army Medical University and analyzed by flow cytometry (CytoFLEX LX, Beckman Coulter). Flow cytometry sorting (MoFlo Astrios EQ, Beckman Coulter) was used to sort the CD8^+^ T lymphocytes from 20 patient-control pairs. The Third International Consensus Definitions for sepsis and septic shock (Sepsis-3.0) were used as the criteria for sepsis (49). Patient confidentiality was preserved in accordance with the guidelines of the Declaration of Helsinki. The exclusion criteria were as follows: (a) patients younger than 18 years and (b) patients who had preexisting cardiovascular, respiratory, renal, hepatic, immunological, or hematological diseases.

### Animals.

C57BL/6 mice were procured from Hunan Ensiwer Company. All animal experiments were conducted in strict accordance with protocols approved by the Ethics Committee of Army Medical University, ensuring adherence to ethical guidelines for the use of experimental animals. The mice were housed in the animal facility at Daping Hospital of Army Medical University and acclimatized for a period of 1 week prior to the commencement of the experiments. The study utilized both male and female mice, with age- and sex-matched controls (8–12 weeks old) included in each experimental group to ensure the consistency and reliability of the results.

### CLP.

The CLP procedure was conducted following the methodology described by Rittirsch et al. (50). Mice were anesthetized with 3% isoflurane to ensure adequate sedation. A midline abdominal incision was made, and one-third of the distal cecum was ligated using 4–0 silk sutures. The cecum was subsequently punctured once with an 18-gauge needle, allowing a portion of the intestinal contents to extrude through the puncture site. The cecum was then repositioned within the abdominal cavity, and the incision was sutured closed. To facilitate recovery, 1.0 mL of 0.9% normal saline was administered subcutaneously, and the mice were placed in a heat preservation box maintained at 37°C.

### Sepsis second-hit model.

Three days after the CLP surgeries, the surviving mice were administered *Streptococcus pneumoniae* [CMCC(B) 31001] as a second hit to produce sepsis-induced immunosuppression. The mice received a 40 μL suspension of *S*. *pneumoniae* (5 × 10^8^ CFU) via intranasal instillation, ensuring that the bacterial suspension reached the bronchi. The control group comprised mice that underwent the same procedure, except that they received 0.9% normal saline intranasally. The survival rate of the mice was monitored for 7 days after the induction of pneumonia.

### Pharmacological inhibition of HDAC1.

The HDAC1 inhibitor mocetinostat (MGCD0103) was obtained from Selleck Chemicals (S1122). For in vivo administration, mocetinostat was initially dissolved in DMSO (40 mg/mL) and then diluted in a vehicle buffer consisting of saline and Kolliphor EL (C5135; Sigma-Aldrich) at a 4:1 ratio. In the intervention experiments, mice received an i.p. injection of mocetinostat at a dose of 10 mg/kg. Treatment was initiated 1 hour after the second hit (*S*. *pneumoniae* infection). Control mice were administered an equivalent volume of the vehicle (DMSO/saline/Kolliphor).

### Isolation of cells from mouse lungs.

Lungs were collected from 8 mice at different time points and digested into single-cell suspensions using dispase, DNase I, and collagenase I, as previously described (51).

### scRNA-Seq data processing, quality control, and cell clustering.

Sorted cells (viability >90%) were processed using the Chromium Single Cell 3′ Reagent Kit v3.0 (10x Genomics) to capture approximately 10,000 cells. Libraries were sequenced on a NovaSeq 6000 (Illumina). Raw data were processed using Cell Ranger (v2.2.0) for alignment to the mouse reference genome (mm10) and generation of feature-barcode matrices.

The raw gene expression matrices were imported into R using the Seurat package (version 4.0). Ambient RNA contamination was first corrected using the SoupX package. Prior to quality control filtering, potential multiplets were identified and removed using Scrublet. Rigorous quality-control filtering was then applied to the remaining cells using an adaptive outlier detection strategy based on the median absolute deviation (MAD). The percentage of mitochondrial reads (percent.mt) was calculated for each cell based on genes starting with “mt-.” Cells were filtered out if they met any of the following criteria: (a) fewer than 200 detected genes; (b) a number of detected genes (nFeature_RNA) exceeding the median plus 2.5 × MAD; or (c) a mitochondrial percentage exceeding the median plus 2.5 × MAD. The distributions of quality-control metrics after filtering are presented in Supplemental Figure 1.

Gene expression levels for the remaining high-quality singlet cells were normalized using the LogNormalize method with a scale factor of 10,000. We identified the top 2,000 highly variable features using FindVariableFeatures. To eliminate potential batch effects among samples, data integration was performed using the anchor-based integration workflow in Seurat. Subsequently, data were scaled using ScaleData, during which cell-cycle heterogeneity was regressed out. Principal component analysis was performed on the scaled data, and the top 30 principal components were selected for dimensionality reduction. Cell clustering was conducted using the FindNeighbors and FindClusters functions with default parameters (resolution = 0.5).

To visualize the data structure, uniform manifold approximation and projection (UMAP) was generated using the RunUMAP and DimPlot functions. Cluster-specific marker genes were identified using the FindAllMarkers function (Wilcoxon rank-sum test; log_2_ fold change > 0.25; min.pct = 0.25).

### T cell subtype annotation and scoring.

To accurately annotate T cell subtypes and states, we utilized ProjectTILs, a reference-based projection method. Query scRNA-Seq data were projected onto the murine T cell reference atlas to classify CD8^+^ T cells into subtypes, including naive, effector memory, and exhausted populations. The Seurat function “AddModuleScore” includes T cell exhaustion, T cell effector factors, innate immunity, antigen presentation, cellular apoptosis, cellular proliferation, and chemokine- and cytokine-related genes. Each cell was scored on the basis of its expression of the genes within the gene sets.

### Transcription factor network inference.

To identify potential upstream regulators of T cell exhaustion, we performed single-cell regulatory network inference and clustering using SCENIC (specifically the pySCENIC workflow). The coexpression of transcription factors and their target genes was inferred using GRNBoost2, and regulon activity scores were calculated for each cell using the AUCell algorithm with the mm10 motif database.

### Imputation analysis.

To visualize gene-gene relationships and mitigate the effects of technical dropouts inherent to scRNA-Seq data, we applied Markov affinity-based graph imputation of cells (MAGIC) using the Rmagic package. The imputed gene expression matrix was utilized specifically for the correlation analyses between *Hdac1* and exhaustion markers (*Pdcd1*, *Havcr2*, and *Cd244*).

### RNA-Seq.

RNA libraries were prepared from sorted CD8^+^ T cells using the SMART-Seq HT Kit (Takara Bio) and validated by Agilent 2100 Bioanalyzer. Sequencing on an Illumina XTen platform generated an average of 26.6 million (sepsis) and 34.4 million (healthy) clean reads after filtering. Clean reads were mapped to the reference genome, and gene expression quantification and differential analysis were performed using the edgeR R package. Differentially expressed genes were identified based on a fold-change of 1.5 or greater and a *P* value less than 0.05. The state of CD8^+^ T cells was analyzed using the TCellSI package. Functional enrichment analyses, including GO biological processes and Kyoto Encyclopedia of Genes and Genomes (KEGG) pathways, were conducted using the clusterProfiler R package. The org.Hs.eg.db package was utilized for gene annotation. A *P* value less than 0.05 was considered statistically significant for enrichment terms. Protein-protein interaction analysis was conducted using the STRING database to identify hub genes. The significance of these genes within the network was further evaluated using CytoNCA and cytoHubba calculations.

### Flow cytometry analysis.

Peripheral blood samples from patients with sepsis, healthy volunteers, and mice were subjected to red blood cell lysis immediately after collection. Human peripheral blood was incubated with surface antibodies for 10 minutes at room temperature in the dark. The antibodies used included CD45-Krome Orange (J33, B36294, Beckman Coulter), CD3-APC-A750 (UCHT1, A94680, Beckman Coulter), CD4-FITC (13B8.2, A07750, Beckman Coulter), Percp anti-human CD8 (SK1, BioLegend, 344707), and CD279-APC (PD1.3, B30633, Beckman Coulter). For mouse samples, staining was conducted for 30 minutes at 4°C in the dark with BV510 anti-mouse CD8a (53-6.7, 100752, BioLegend), Alexa Fluor 647 anti-mouse CD279 (RMP1-30, 109118, BioLegend), PerCP5.5 anti-mouse CD244.2 (2B4 B6, 133513, BioLegend), PE anti-mouse CD366 (5D12, 566364, BD Pharmingen), APC/Cy7 rat anti-mouse CD45 (30-F11, 561037, BD Pharmingen), and BV421 anti-mouse CD3 (17A2, 100228, BioLegend) antibodies. After washing, the samples were fixed and permeabilized using a Transcription Factor Buffer Set (562574, BD Pharmingen), followed by intracellular staining. The HDAC1 antibody (EPR460(2), ab09411, Abcam) was incubated on ice for 30 minutes, washed, and subsequently incubated with goat anti-rabbit IgG H&L (Alexa Fluor 488) for 30 minutes in the dark. Isotype control IgGs were used as controls. After 2 washes, the cells were resuspended for analysis. For apoptosis staining, the cells were initially incubated with Zombie UV dye diluted 1:1,000 in PBS for 10 minutes in the dark prior to surface antibody staining. After the samples were washed and resuspended in binding buffer, Annexin-PE dye (Beyotime, C1065S) was added, and the samples were incubated for 25 minutes in the dark. The cells were then washed, resuspended, and analyzed using a CytoFLEX LX flow cytometer (Beckman Coulter). Data analysis was performed using FlowJo software (version 10.8.1, BD Biosciences), and the gating strategy is shown in Supplemental Figure 7.

### Transient transfection.

Jurkat E6-1 T cells from the American Type Culture Collection (ATCC) grown in RPMI 1640 (Gibco) supplemented with 10% FBS, 2 mmol/L glutamine, 2 mmol/L penicillin, and streptomycin were transiently transfected with the CALNP RNAi kit (D-Nano Therapeutics) according to the manufacturer’s protocol. In brief, 5 × 10^5^ cells were resuspended in 100 μL of RPMI 1640 containing 10% FBS and mixed with 20 μmol of siRNA. The following *HDAC1*-mutant siRNAs were purchased from GenePharma: si-1308: 5′- GCUCCUCUGACAAACGAAUTT -3′ (sense), 5′- AUUCGUUUGUCAGAGGAGCTT -3′ (antisense); si-595: 5′-GCUGUACAUUGACAUUGAUTT-3′ (sense), 5′- AUCAAUGUCAAUGUACAGCTT -3′ (antisense); si-1122: 5′- GUCCUUCCAAUAUGACUAATT -3′ (sense), 5′- UUAGUCAUAUUGGAAGGACTT -3′ (antisense).

### ChIP assay.

ChIP assays were performed according to the kit protocol (CST, 9003). Briefly, isolated chromatin was immunoprecipitated using an anti-NFAT1 (D43B1, CST, 5861) antibody, anti-HDAC1 antibody (D5C6U, CST, 34589), anti-Ac-H3 antibody, or normal rabbit IgG (negative control). The immunoprecipitated DNA was analyzed using real-time PCR. For quantitative ChIP, amplification was performed using GoTaq Probe qPCR master mix (Promega). The data were analyzed with the percent input method: 2% × 2^(C[T]^ ^Input^ ^Sample^^–^^C[T]^ ^IP^ ^Sample)^. The primers used for amplifying the *Pdcd1* gene were 5′-TAAACGAGAGCTTCCTCGCC -3′ (forward) and 5′-CCGGCTCTGAAGGGAAAACA -3′ (reverse).

### CD8^+^ T cell isolation and adoptive transfer.

CD8^+^ T cells were isolated from the spleens of healthy WT mice using magnetic-activated cell sorting. Briefly, spleens were harvested and mechanically dissociated through a 70-μm cell strainer to prepare single-cell suspensions. After RBC lysis (5 minutes at room temperature), cells were washed and resuspended in PBS containing 0.5% BSA and 2 mM EDTA. CD8^+^ T cells were then positively selected using CD8 (Ly-2) MicroBeads (Miltenyi Biotec; 130-045-201) according to the manufacturer’s instructions. The purity of the enriched CD8^+^ T cell population was verified by flow cytometry to be greater than 92% (Supplemental Figure 2B). For adoptive transfer experiments, the purified cells were centrifuged at 350*g* for 5 minutes and resuspended in PBS to a final concentration of 5 × 10^6^ cells/mL. In the CLP-induced polymicrobial sepsis model, mice were administered 5 × 10^5^ cells via tail vein injection (100 μL/mouse) 1 hour after the secondary insult. Serum biochemistry and immunophenotyping via flow cytometry were conducted 3 days after cell transfer, and on day 4, major organs were harvested.

### T cell in vitro stimulation.

Freshly isolated lymphocytes were seeded in 6-well plates (Corning) at a concentration of 2 × 10^6^ cells/well in 3 mL of complete culture medium. The medium consisted of RPMI 1640 (Gibco) supplemented with 10% FBS, 1% penicillin-streptomycin (HyClone Laboratories, Inc.), and 1% L-glutamine (HyClone Laboratories, Inc.). To simulate chronic stimulation, the cells were cultured for 14 days in a humidified CO_2_ incubator at 37°C in complete culture medium supplemented with purified anti-human CD3 antibodies (OKT3, 317320, BioLegend) at 1 μg/mL, purified anti-human CD28 antibodies (CD28.2, 302902, BioLegend) at 1 μg/mL, and recombinant human IL-2 (rhIL-2; 25 U/mL; Proteintech). The cells were harvested on days 1, 3, and 14 for flow cytometry analysis and CD8^+^ T cell isolation.

### Statistics.

Statistical analyses were performed using R software (version 4.5.0) and GraphPad Prism 10. Continuous data are expressed as mean ± SD. Data distribution normality was assessed using the Shapiro-Wilk test. Data that passed the normality test were then assessed by unpaired 2-tailed *t* tests (2 groups) or 1-way ANOVA with Tukey’s test (3 groups or more), where indicated. For non-normally distributed data or datasets with small sample sizes, nonparametric tests were applied, including the Mann-Whitney *U* test (2 groups) or the Kruskal-Wallis test followed by Dunn’s multiple-comparison test (multiple groups).

Survival analysis was conducted using Kaplan-Meier curves, with significance determined by the log-rank test. Cox proportional hazards models were utilized to evaluate time-to-event outcomes. To assess associations between clinical characteristics and immune cell subsets, multivariable linear regression models were constructed using R, adjusting for potential confounders such as age. The nonlinear relationship between lymphocyte percentage and mortality was modeled using restricted cubic spline regression.

Differential gene expression for single-cell and bulk RNA-Seq was analyzed using the Wilcoxon rank-sum test (via Seurat) and generalized linear models (via edgeR), respectively, as detailed in the bioinformatic analysis subsections. A *P* value of less than 0.05 was considered statistically significant.

### Study approval.

This study protocol was approved by the Ethics and Protocol Review Committee of the Army Medical University (TMMU2020009). Sample collection was performed with the approval of the Ethics Committee of Daping Hospital of Army Medical University (ClinicalTrials.gov NCT01713205). Written informed consent was obtained from all patients or their next of kin.

### Data availability.

All data points shown in graphs and values behind reported means are provided in the Supporting Data Values XLS file. The RNA-Seq data generated in this study (human bulk RNA-Seq and mouse scRNA-Seq) have been deposited in the OMIX, China National Center for Bioinformation/Beijing Institute of Genomics, Chinese Academy of Sciences (https://ngdc.cncb.ac.cn/omix, under accession numbers OMIX013472 and OMIX013473).

Detailed experimental protocols for scRNA-Seq library preparation and raw data processing, RT-qPCR, histological assessment (H&E), immunofluorescence staining, and Western blotting are provided in the Supplemental Methods.

## Author contributions

DL was the primary researcher for this study. JBF, RW, WDB, SYH, HHN, XFF, HCZ, XFH, ZX, YL, and JD were involved in the collection of blood samples and clinical data. FX, SSD, and JXJ revised the manuscript. AYY contributed to data analysis. XX, ZW, and LZ planned the study, and LZ wrote the manuscript. All the authors have read and approved the final manuscript.

## Funding support

- The National Natural Science Foundation of China (82222038 to LZ, 82502994 to DL, and 81970259 to ZW).
- The Outstanding Young Talents of National Defense Biotechnology (2023-JCJQ-ZQ-001 and 01-SWKJYCJJ06 to LZ).
- The Chongqing Outstanding Youth Fund (CSTB2022NSCQ-JQX0017 to LZ).
- The Military Clinical Key Specialty Construction Project (to ZW).
- The Special Project for Enhancing Technological Innovation Capacity of Army Medical University (2023XQN49 to DL).

## Supplementary Material

Supplemental data

Unedited blot and gel images

Supporting data values

## Figures and Tables

**Table 1:**
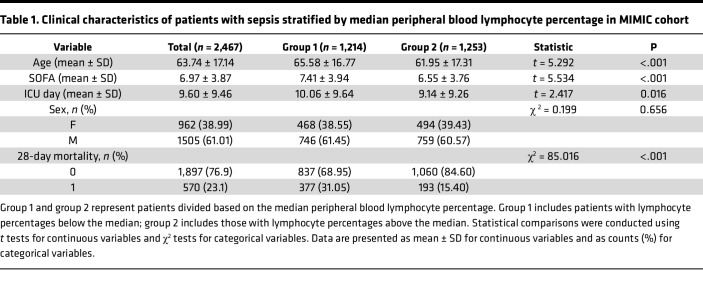
Clinical characteristics of patients with sepsis stratified by median peripheral blood lymphocyte percentage in MIMIC cohort

## Version 1 02/23/2026

Electronic publication
