# Supplementary material for: HDAC1 modulates sepsis-induced immunosuppression by driving the exhaustion of CD8+ T cells
Source: JCI Insight. 2026 Feb 23;11(4):e197224. doi: 10.1172/jci.insight.197224 (PMC13067949; doi:10.1172/jci.insight.197224)
Supplement: Supplemental data [file jciinsight-11-197224-s159.pdf]

HDAC1 modulates sepsis-induced immunosuppression by driving the exhaustion of $CD8^+$ T cells

Di Liu, Jiangbo Fan, Rui Wang, You Li, Wanda Bi, Siyuan Huang, Henghai Nie, Xifeng Feng, Huacai Zhang, Juan Du, Xiaofei Huang, Anyong Yu, Zhe Xu, Fei Xia, Jianxin Jiang, Shuangshuang Dai, Xiang Xu, Zhen Wang, Ling Zeng

Supplementary materials and methods 2
Table S1 5
Table S2 6
Table S3 6
Figure S1 7
Figure S2. 9
Figure S3 11
Figure S4 12
Figure S5 13
Figure S6 15
Figure S7. 16

Supplementary materials and methods

Single-cell RNA-seq library preparation and raw data processing

Single cells were collected in RPMI 1640 containing 5% FBS, and viability was confirmed to be > 90% using Trypan blue exclusion. The sorted cells were counted and resuspended at a concentration of 700-1200 cells/µl. Single-cell suspensions were subsequently loaded onto a 10x Chromium to capture approximately 10,000 single cells using Chromium Single Cell 3' Reagent kits v3.0 (10× Genomics, Pleasanton, CA, USA). The cells were partitioned into gel beads in the Chromium instrument, where cell lysis and barcoded reverse transcription of RNA occurred. DNA amplification and library construction were then performed. Libraries were sequenced on a NovaSeq 6000 (Illumina) by C-Bio Technology. The raw scRNA-seq data were processed using Cell Ranger (version 2.2.0) to demultiplex the raw data and generate FASTQ files, align the reads to the mouse reference genome (mm10), count barcodes and unique molecular identifiers (UMIs), and generate feature-barcode matrices.

RT–PCR

The analysis of RNA extracted using TRIzol (Invitrogen) and CHCl3 involved the synthesis of complementary DNA (cDNA) from one microgram of RNA using the GoScript cDNA Synthesis Kit (#Promega, # Z6010). Quantitative real-time PCR (qRT–PCR) was then performed with the synthesized cDNA, specific primers, and SsoFast EvaGreen Supermix (#172–5204, Bio-Rad). The expression of target genes was quantified using the comparative Ct (ddCt) method, with normalization to the expression of the housekeeping gene *GAPDH*. The data are presented as the relative quantity (RQ), with the RQ of the control cells set to one for comparative purposes.

Hematoxylin and eosin staining

Lungs were fixed with 4% paraformaldehyde in phosphate-buffered saline, dehydrated through a series of graded ethanol solutions (75%, 80%, 90%, 95%, and 100%) and cleared in xylene to prepare them for paraffin embedding. Once they were embedded in paraffin, the tissues were sectioned into 4-micron-thick slices using a microtome and mounted onto microscope slides. For staining, the sections were first immersed in hematoxylin to stain the nuclei, followed by differentiation in acidic alcohol to remove nonspecific staining. The sections were subsequently stained with eosin to color the cytoplasm and extracellular matrix. After staining, the sections were dehydrated again using a graded ethanol series, cleared in xylene, and mounted with a coverslip using neutral resins.

Immunofluorescence and confocal microscopy

For the analysis of NFAT1 nuclear translocation and FRET assays, sorted $CD8^+$ T cells were seeded onto poly-L-lysine-coated slides, fixed with 4% paraformaldehyde for 15 minutes, and permeabilized with 0.1% Triton X-100. Cells were blocked and incubated overnight with anti-HDAC1 (10E2, CST, #5356) and anti-NFAT1 (D43B1, CST, #5861) antibodies, followed by incubation with Alexa Fluor 488- and Alexa Fluor 405-conjugated secondary antibodies. Nuclei were counterstained with DAPI. Images were acquired using a Leica Stellaris 5 confocal microscope. For FRET analysis, the acceptor photobleaching method was employed. The FRET efficiency was calculated using the formula: Efficiency = (Dpost - Dpre) / Dpost × 100, where Dpre and Dpost represent the donor fluorescence intensity before and after acceptor photobleaching, respectively. Image analysis was performed using LAS X software (Leica Microsystems).

Western blotting

Total protein was extracted from isolated $CD8^+$ T cells or cultured cells using RIPA lysis buffer (Invitrogen, #FNN0021) supplemented with protease and phosphatase

inhibitor cocktails (Promega, #78440). Protein concentration was determined using a BCA Protein Assay Kit (Beyotime, B0009). Equal amounts of protein (20–30 μg) were separated by 10% SDS-PAGE and transferred onto PVDF membranes (Millipore). The membranes were blocked with 5% non-fat milk in TBST for 1 hour at room temperature and then incubated overnight at 4°C with the following primary antibodies: anti-HDAC1 (D5C6U, CST, #34589), anti-c-Fos (E217R, CST, #31254), anti-c-Jun (60A8, CST, #9165), anti-phospho-c-Fos (D82C12, CST, #5348), anti-phospho-c-Jun (D47G9, CST, #3270), and anti-GAPDH (D16H11, CST, #5174). After washing with TBST, the membranes were incubated with HRP-conjugated secondary antibodies (CST, #7076) for 1 hour at room temperature. The blots were analyzed using a ChemiDoc Touch Imaging System (Bio-Rad, USA). Image Lab software was used to analyze the band intensities.

Table S1. Clinical characteristics of sepsis patients included in the prospective study

	n=140
Age (yrs)	60.76±14.80
Sex (male/female), n	100/40
**Number of dysregulated organs, n (%)**	
1	64 (60.7)
2	40 (28.6)
3 or more	15 (10.7)
**Source of Infection, n (%)**	
Respiratory tract	64 (45.7)
Blood	5 (3.6)
Urinary tract	10 (7.1)
Digestive system	40 (28.6)
Mixed	13 (9.3)
Trauma	8 (5.7)
**Pathogens, n (%) (Blood culture positive)**	
Gram's negative bacteria	49
Gram's positive bacteria	24
Fungus	7
Mixed infection of Gram's positive and negative bacteria	27
virus	33
SOFA score	6.8±4.1
ICU stay (days)	19.59±17.9
28-day mortality, n (%)	32 (22.9)

Table S2. Mediation analysis for CD8$^+$ T-cell percentage, SOFA score, and ICU mortality

Effect	Estimate	Lower	Upper	*β* (95%CI)	*P*	Mediation (%)
Indirect	-0.014	-0.024	-0.003	-0.014 (-0.024 ~ -0.003)	**<.001**	82.317
Direct	-0.001	-0.010	0.012	-0.001 (-0.010 ~ 0.012)	0.618	17.683
Total	-0.015	-0.021	-0.005	-0.015 (-0.021 ~ -0.005)	**0.014**	100.000

Adjustment: Age.

Model: quasi-Bayesian approach, 1000 simulations

Table S3. Mediation analysis for CD4$^+$ T-cell percentage, SOFA score, and ICU mortality

Effect	Estimate	Lower	Upper	β (95% CI)	P	Mediation (%)
Indirect	0.001	-0.002	0.004	0.001 (-0.002 ~ 0.004)	0.306	37.304
Direct	-0.000	-0.007	0.003	-0.000 (-0.007 ~ 0.003)	0.766	62.696
Total	0.001	-0.006	0.003	0.001 (-0.006 ~ 0.003)	0.466	100.000

Adjustment: Age.

Model: quasi-Bayesian approach, 1000 simulations

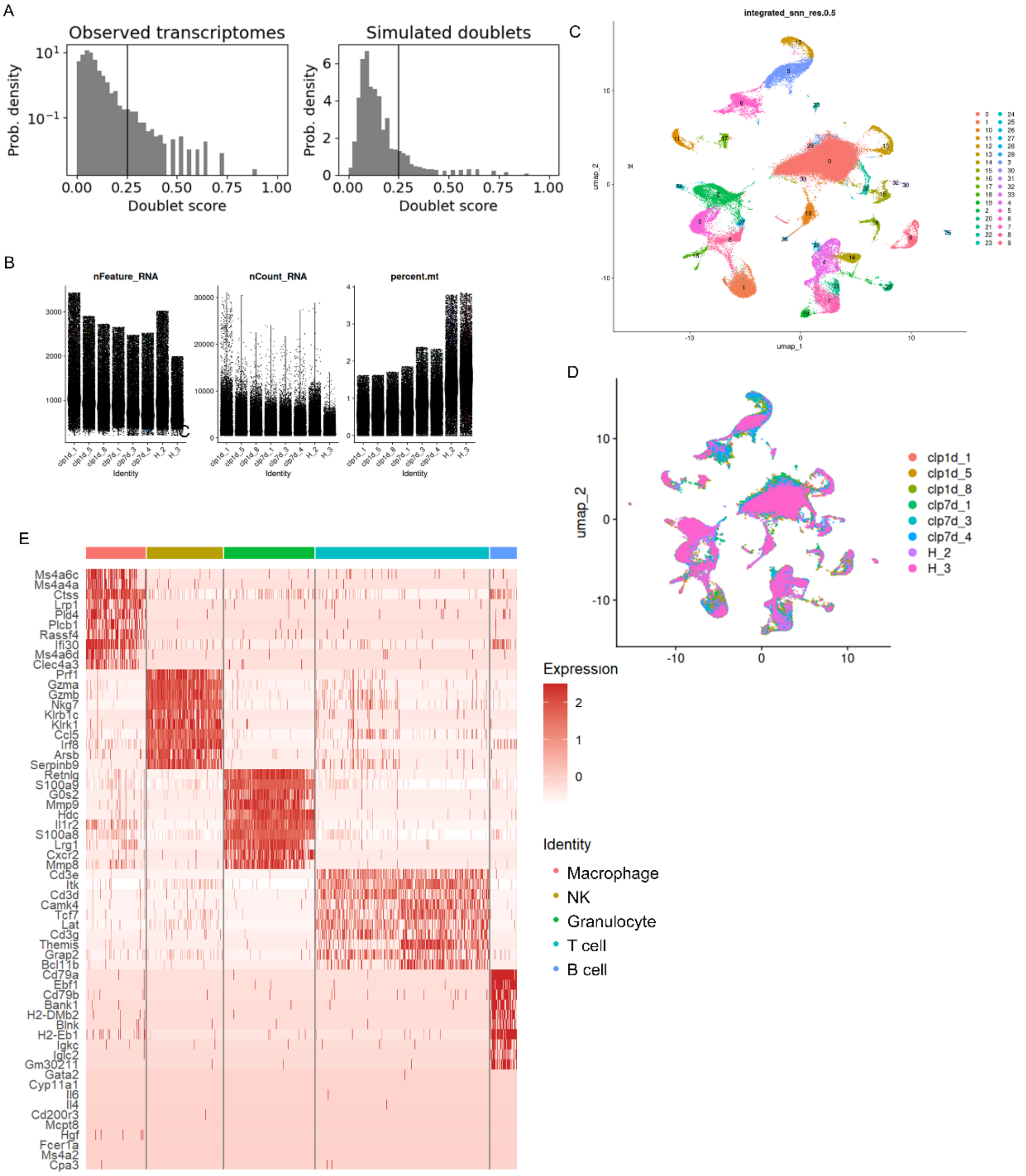

Figure S1. Quality control, doublet removal, and clustering analysis of scRNA-seq data. (**A**) Doublet detection and removal using Scrublet. Histograms display the distribution of observed (left) and simulated (right) doublet scores. The vertical line at 0.25 indicates the threshold used to identify doublets; cells with scores to the right of this threshold were excluded from downstream analysis. Y-axis represents the density of cells. (**B**) Violin plots showing post-filtering quality control metrics for each sample. Metrics include the number of detected genes (*nFeature_RNA*), unique molecular identifiers (*nCount_RNA*), and the percentage of mitochondrial reads (*percent.mt*). (**C** and **D**) Uniform Manifold Approximation and Projection (UMAP) plots of the

integrated dataset. (**C**) Unbiased clustering of all analyzed pulmonary cells. (**D**) UMAP plots split by each sample (Control, CLP 1d, and CLP 7d), demonstrating the distribution of cells from each condition. (**E**) Heatmap displaying the expression levels of the top 10 marker genes for each identified cluster. Key exemplar genes and assigned cell-type annotations are labeled on the right. Red indicates high expression, while white indicates low expression.

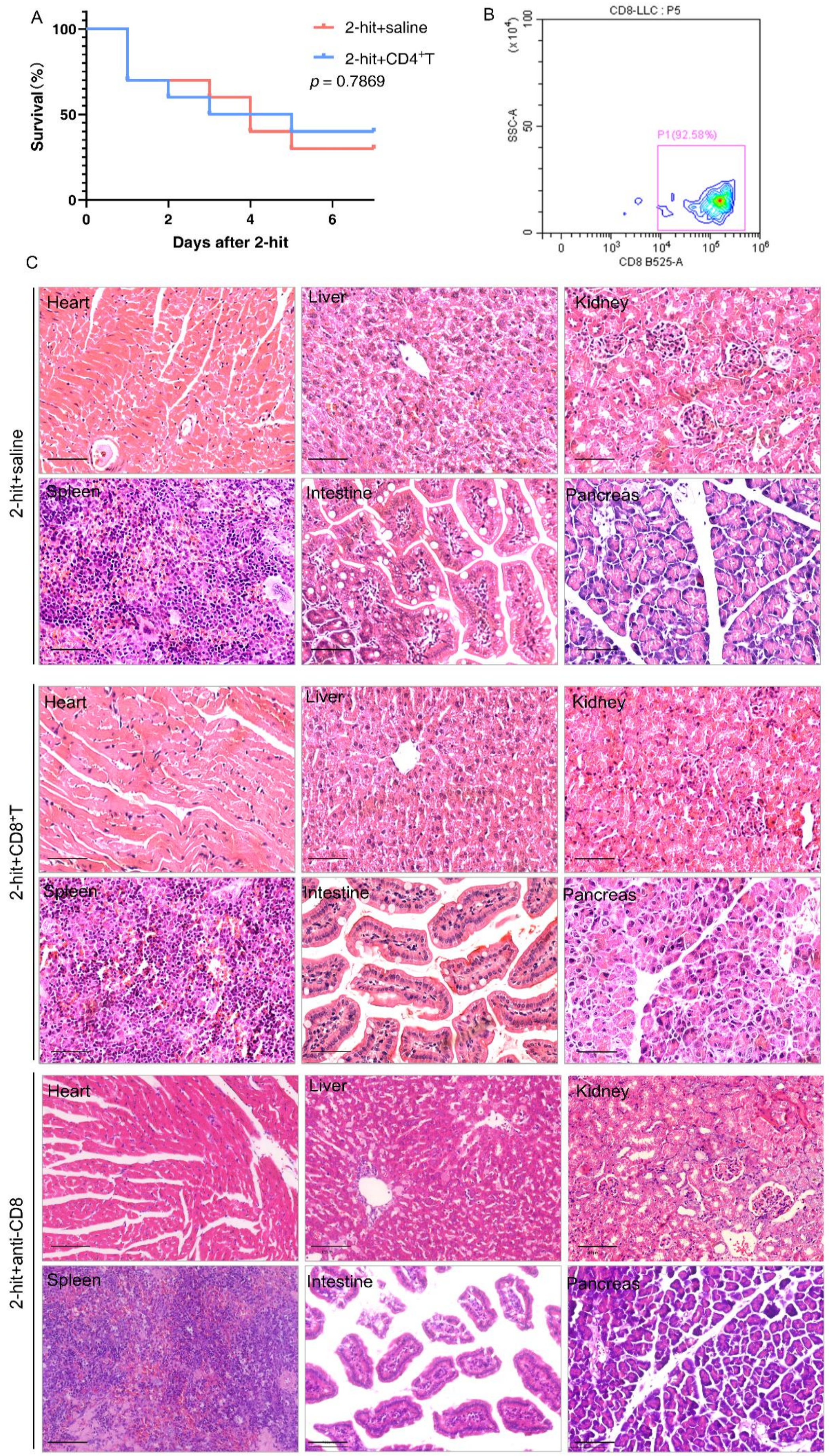

A
Survival (%)
100
50
0
0
2
4
6
Days after 2-hit
2-hit+saline
2-hit+CD4$^+$T
p = 0.7869
B
CD8-LLC : P5
(x 10^4)
SSC-A
P1(92.58%)
CD8 B525-A
C
2-hit+saline
2-hit+CD8$^+$T
2-hit+anti-CD8
Heart
Liver
Kidney
Spleen
Intestine
Pancreas

Figure S2. The effect of $CD4^+$ T and $CD8^+$ T cells on the mortality rate of sepsis. (**A**) The effect of $CD4^+$ T cells transfer on the survival rate of 2-hit model (P = 0.7869). (**B**) Representative flow cytometry plots of $CD8^+$ T cell purity following MACS isolation. The isolated population demonstrated 92.58% purity. (**C**) Representative H&E-stained histological sections of heart, liver, kidney, spleen, small intestine, and pancreas of the 2-hit mice, 2-hit model with $CD8^+$ T cells transfer (5×10^5 cells per mouse), 2-hit model with anti-CD8 antibody treatment. Scale bars = 100 μm.

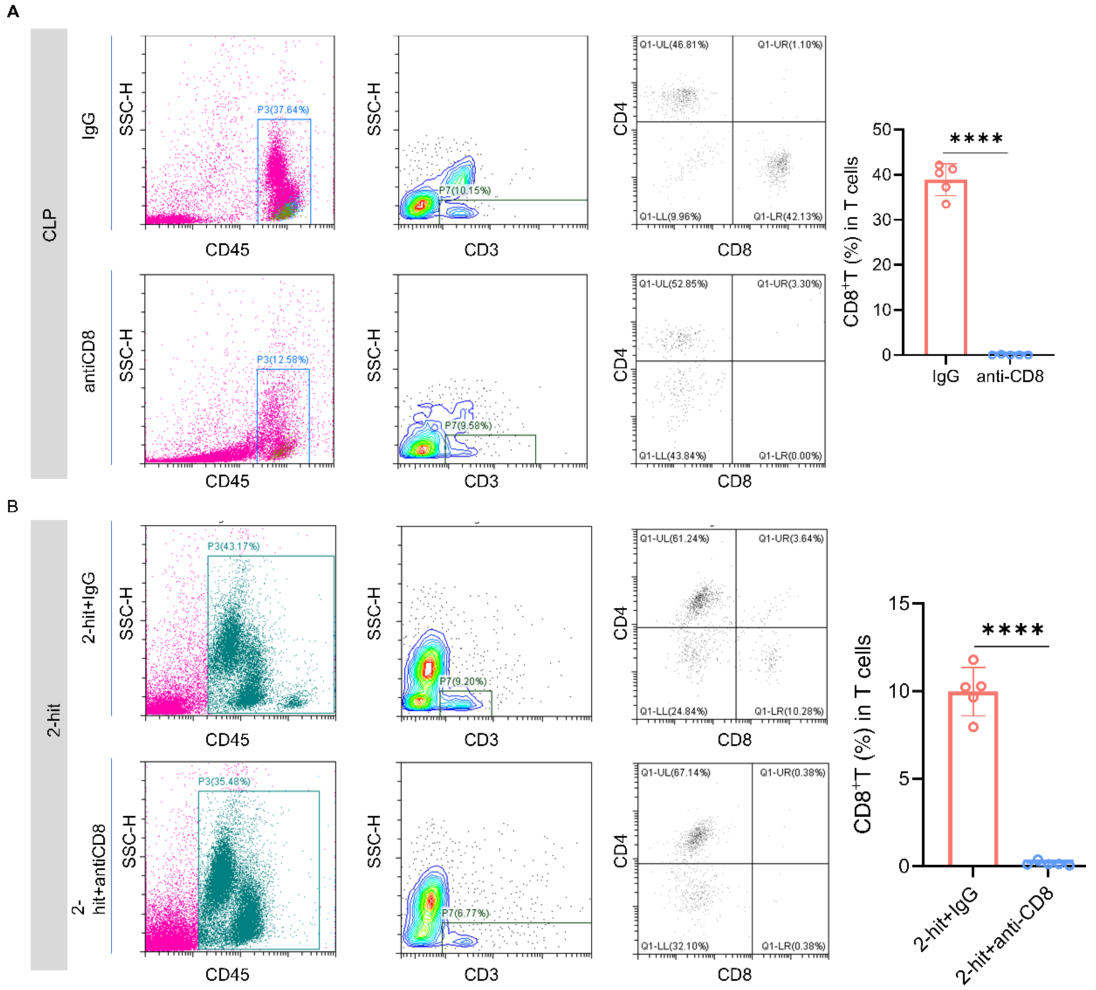

Figure S3. Flow cytometry analysis of CD8^{+} T cell depletion by administration of anti-CD8 monoclonal antibody (mAb). (**A**) Representative flow cytometry plots of peripheral blood CD8^{+} T cells from mice treated with IgG or anti-CD8 antibody prior to the second hit in the 2-hit model. The bar chart illustrates the percentage of CD8^{+} T cells of the IgG control and anti-CD8 mAb treatment groups. (**B**) Representative flow cytometry plots of peripheral blood and CD8^{+} T cells from mice treated with IgG or anti-CD8 mAb at the end of the observation period in the 2-hit model. The bar chart illustrates the percentage of CD8^{+} T cells of the IgG control and anti-CD8 mAb treatment groups.

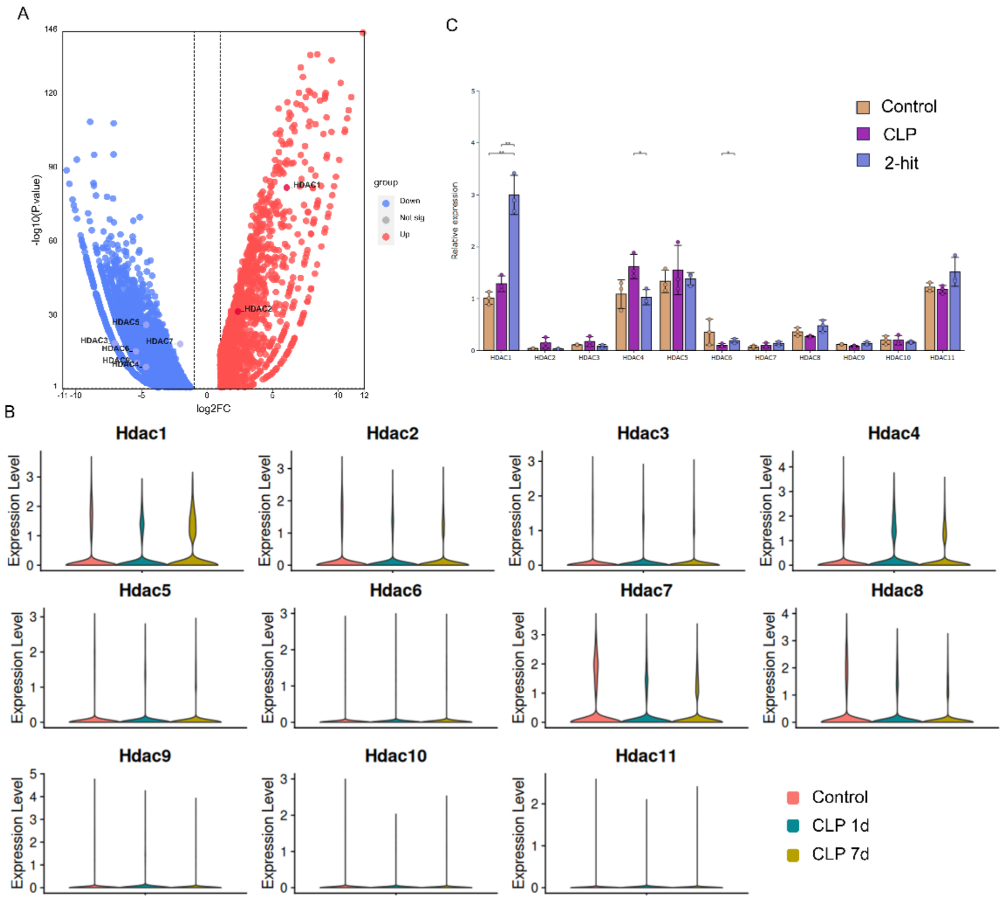

Figure S4 Specificity of HDAC1 upregulation compared with other HDAC family members. (**A**) Volcano plot displaying the differential expression of HDAC family members in human peripheral blood CD8^{+} T cells (Sepsis vs. Healthy). *HDAC1* and *HDAC2* are significantly upregulated (red) ($P < 0.0001$), whereas other isoforms (*HDAC3–9*) are downregulated (blue). (**B**) Single-cell expression dynamics of *Hdac1–11* in murine lung CD8^{+} T cells across different groups (Control, CLP 1d, and CLP 7d). (**C**) Quantitative PCR validation of *Hdac1–11* mRNA levels in splenic CD8^{+} T cells from Control, CLP, and 2-hit model mice. Data are presented as mean ± SD. Statistical significance was determined by differential expression analysis (A) or one-way ANOVA followed by Tukey's post hoc test (C). *P* values indicate statistical significance. *$P < 0.05$, **$P < 0.01$.

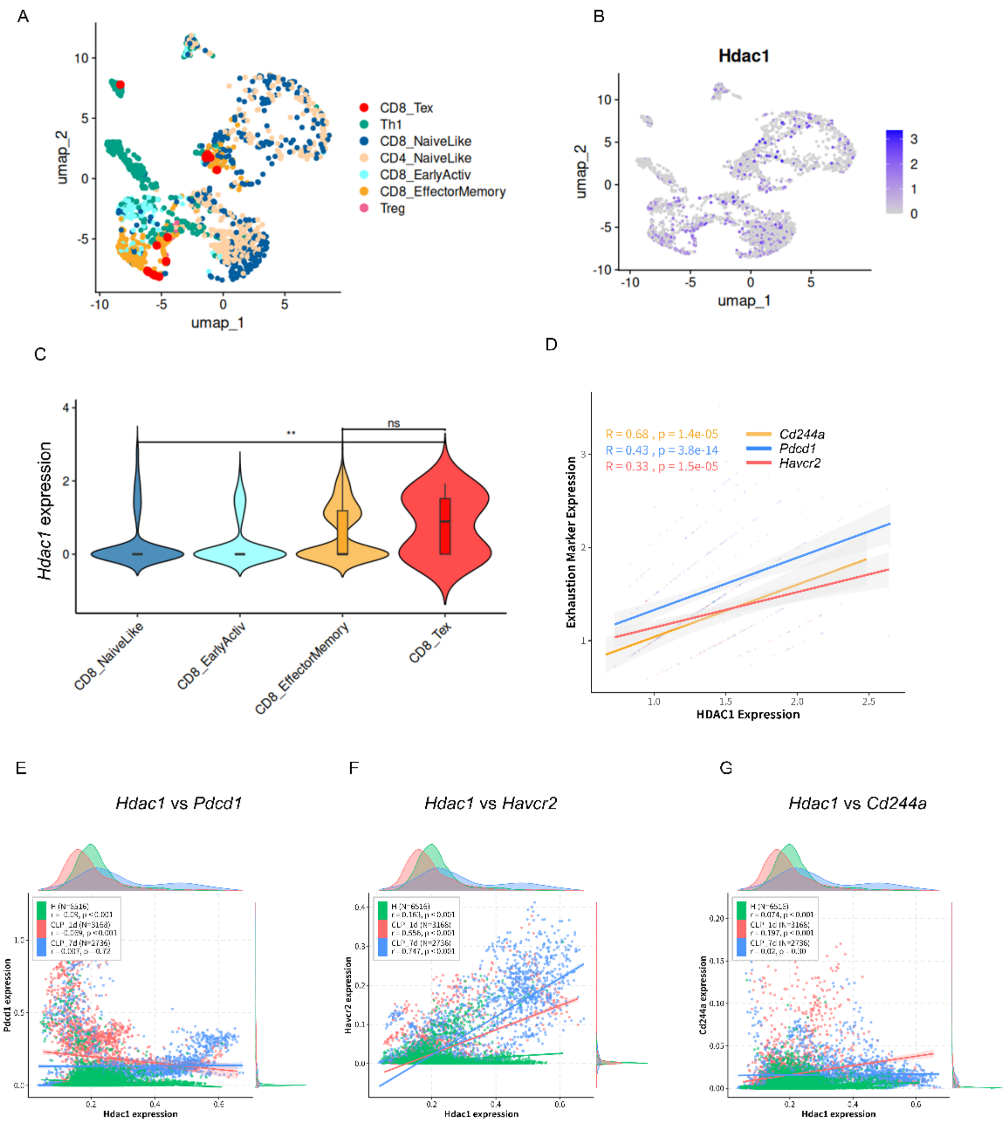

Figure S5 Single-cell analysis reveals the specific association of HDAC1 with terminal exhaustion. (**A**) UMAP projection showing the annotated CD8^{+} T-cell subsets (CD8_NaiveLike, CD8_EarlyActiv, CD8_EffectorMemory, and CD8_Tex). (**B** and **C**) Single-cell gene expression analysis of *Hdac1*. (**B**) Feature plot showing the distribution of *Hdac1* expression on the UMAP projection. (**C**) Violin plot displaying *Hdac1* expression levels across distinct CD8^{+} T-cell subsets. Points represent individual cells. Statistical significance was determined by Wilcoxon test (****P* < 0.001, *****P* < 0.0001 compared with the CD8_Tex group). (**D**) Global gene-gene correlation analysis using raw gene expression counts. Scatter plots illustrate a general positive association between *Hdac1* and the exhaustion markers *Pdcd1*, *Havcr2*, and *Cd244* across the

entire $CD8^+$ T-cell population. (**E-G**) Refined correlation analyses using MAGIC-imputed data to mitigate technical dropout effects and resolve relationships across distinct disease states. Scatter plots with marginal density distributions display the correlation between *Hdac1* and (**E**) *Havcr2* (TIM-3), (**F**) *Pdcd1* (PD-1), and (**G**) *Cd244* (2B4). Marginal density plots (top and right panels) accompany each scatter plot, illustrating the expression distribution of the corresponding genes for each experimental group. Pearson correlation coefficients (*R*) and *P* values are indicated.

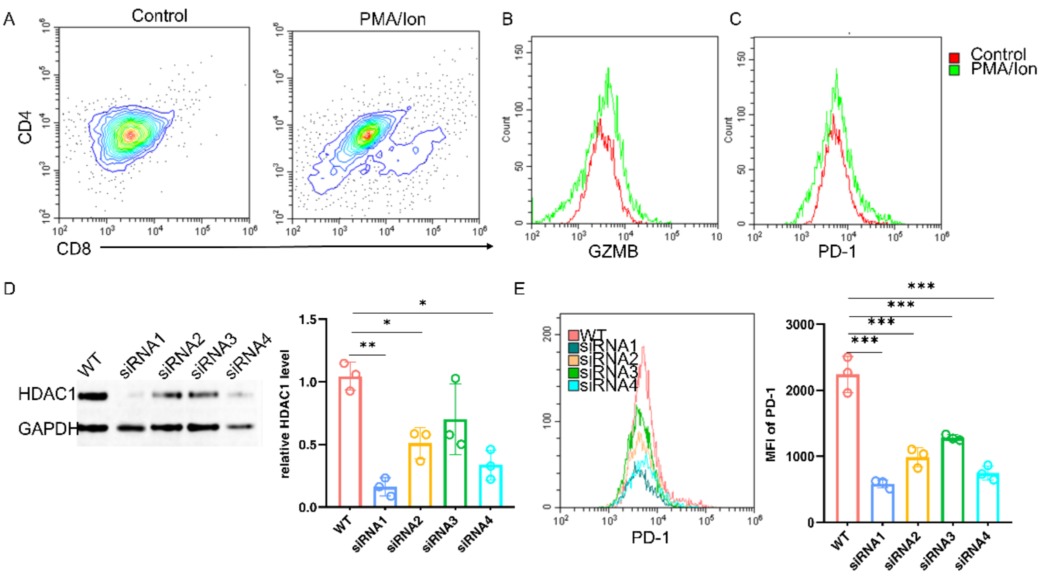

Figure S6. HDAC1 regulates PD-1 expression in activated Jurkat T cells. (**A**) Representative flow cytometry plots showing CD4 and CD8 expression in control and PMA/ionomycin-stimulated Jurkat cells. (**B-C**) Histograms showing increased expression of granzyme B (GZMB, B) and PD-1 (C) in PMA/ionomycin-stimulated Jurkat cells (green) compared to unstimulated controls (red). (**D**) HDAC1 expression in wild-type (WT) and HDAC1 siRNA-transfected Jurkat cells. Left: representative flow cytometry histograms; Right: quantification of HDAC1-positive CD8$^+$ T cells (n=3 per group). ***P* < 0.01, ****P* < 0.001. (**E**) PD-1 expression in WT and HDAC1 siRNA-transfected Jurkat cells. Left: representative flow cytometry histograms; Right: quantification of PD-1 mean fluorescence intensity (MFI) (n=3 per group). ****P* < 0.001. (**F**) DNA electrophoresis showing genomic DNA integrity of WT and siRNA-transfected cells prior to ChIP experiments. (**G**) Western blot confirming appropriate sample quality of WT and siRNA-transfected cells prior to ChIP experiments. (**H**) Standard curve for PDCD1 promoter primers used in ChIP-qPCR analysis ($R^2 = 0.937$, slope = -3.200).

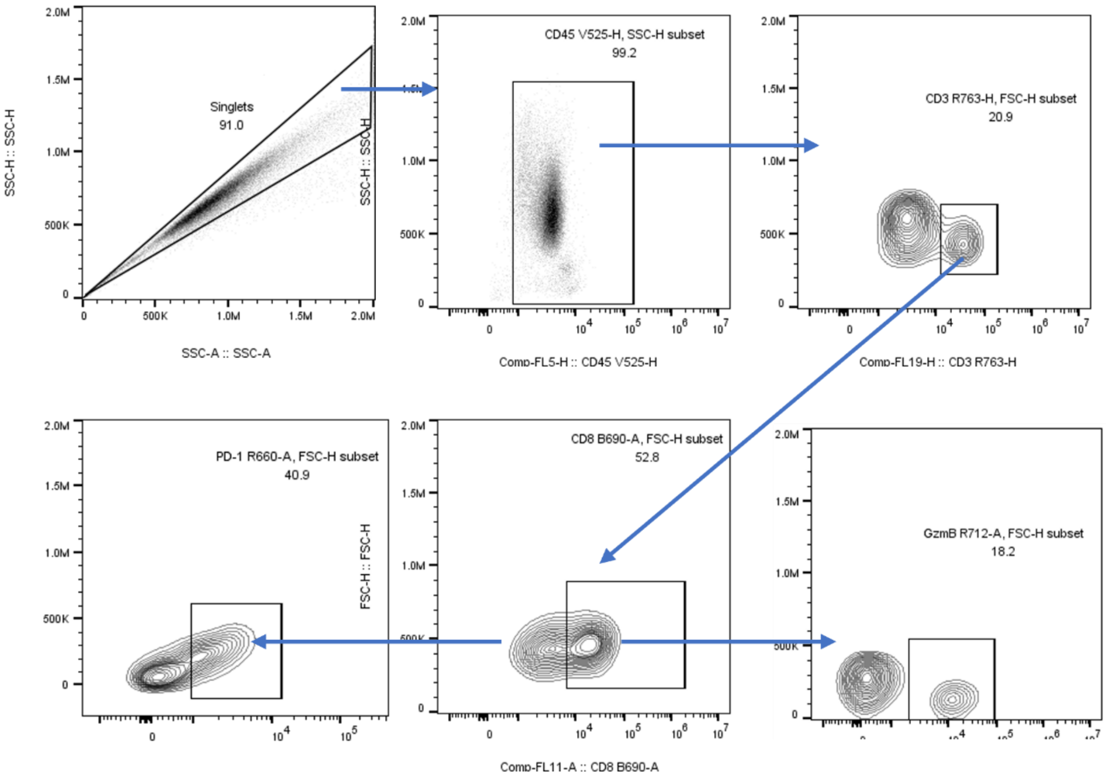

Figure S7. Gating strategy for identification of CD8⁺ T cells, PD-1 and GZMB expression. Flow cytometric analysis was performed on peripheral blood mononuclear cells (PBMCs). Singlets were first gated based on FSC-A and SSC-A to exclude doublets. $CD45^+$ leukocytes were selected, followed by gating on $CD3^+$ T cells. Within $CD3^+$ cells, $CD8^+$ T cells were identified. Expression of PD-1 and Granzyme B (GZMB) was then assessed within the $CD8^+$ T-cell population. Percentages in each gate represent the proportion of cells within the parent population.
