# Supplementary figures and images for: HDAC1 modulates sepsis-induced immunosuppression by driving the exhaustion of CD8+ T cells
Source: JCI Insight. 2026 Feb 23;11(4):e197224. doi: 10.1172/jci.insight.197224 (PMC13067949; doi:10.1172/jci.insight.197224)

The blots outlined in red are the ones presented in the manuscript

Figure 4F

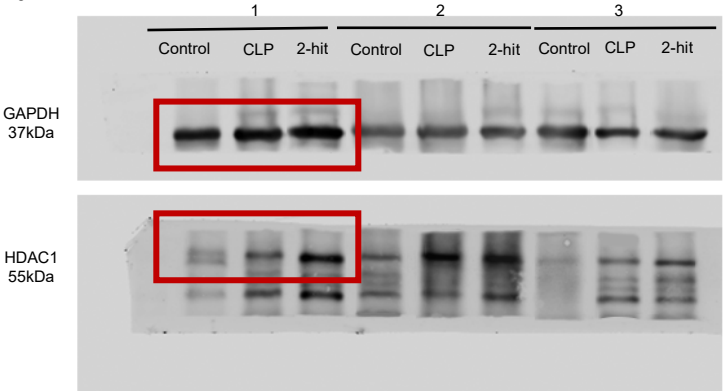

Figure 4G

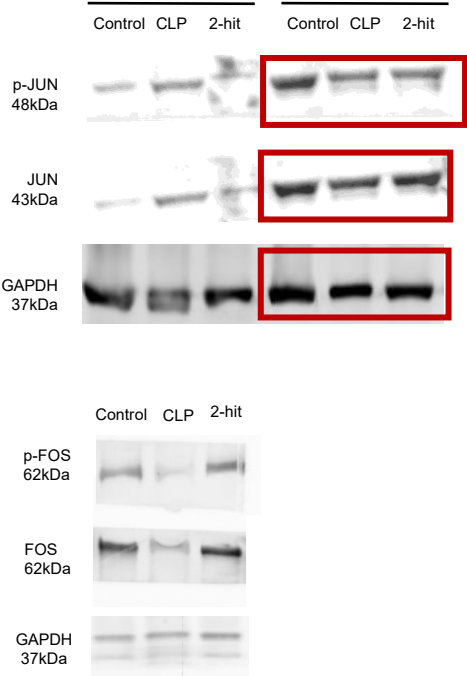

Figure 9D

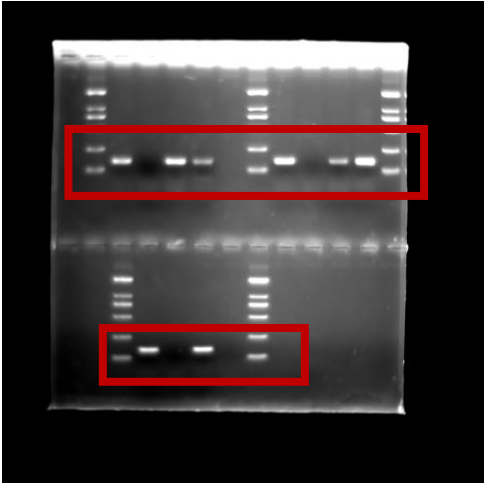

Supplement: Unedited blot and gel images [file jciinsight-11-197224-s160.pdf]
